# Transcriptomic analysis of early stages of ‘*Candidatus* Liberibacter asiaticus’ infection in susceptible and resistant species after inoculation by *Diaphorina citri* feeding on young shoots

**DOI:** 10.3389/fpls.2025.1502953

**Published:** 2025-02-20

**Authors:** Mônica N. Alves, Juan Cifuentes-Arenas, Regina Niñoles, Laudecir Lemos Raiol-Junior, Everton Carvalho, Isabel Quirós-Rodriguez, Jesus A. Ferro, Concetta Licciardello, Berta Alquezar, Lourdes Carmona, Javier Forment, Aureliano Bombarely, Nelson A. Wulff, Leandro Peña, José Gadea

**Affiliations:** ^1^ Universidade Estadual Paulista (Unesp), Faculdade de Ciências Agrárias e Veterinárias (FCAV), Jaboticabal, SP, Brazil; ^2^ Fundo de Defesa da Citricultura (Fundecitrus), Araraquara, SP, Brazil; ^3^ Instituto de Biologia Molecular y Celular de Plantas (IBMCP), Universidad Politécnica de Valencia (UPV-CSIC), Valencia, Spain; ^4^ Helix Sementes e Biotecnologia, Patos de Minas, MG, Brazil; ^5^ Research Center for Olive Fruit and Citrus Crops, Council for Agricultural Research and Economics, Acireale, Italy

**Keywords:** *Citrus*, *Murraya*, *Bergera*, HLB, transcriptomics, Liberibacter

## Abstract

Huanglongbing (HLB) is a devastating disease of citrus plants caused by the non-culturable phloem-inhabiting bacterium *Candidatus* Liberibacter ssp., being *Ca*. Liberibacter asiaticus (*C*Las) the most aggressive species. *C*Las is vectored by the psyllid *Diaphorina citri* and introduced into sieve cells, establishing a successful infection in all *Citrus* species. Partial or complete resistance has been documented in the distant relatives *Murraya paniculata* and *Bergera koenigii*, respectively, providing excellent systems to investigate the molecular basis of HLB-resistance. It has been shown previously that the first weeks after bacterial release into the phloem are critical for the establishment of the bacterium. In this study, a thorough transcriptomic analysis of young flushes exposed to *C*Las-positive and negative psyllids has been performed in *Citrus × sinensis*, as well as in the aforementioned resistant species, along the first eight weeks after exposure. Our results indicate that the resistant species do not deploy a classical immunity response upon *C*Las recognition. Instead, transcriptome changes are scarce and only a few genes are differentially expressed when flushes exposed to *C*Las-positive and negative psyllid are compared. Functional analysis suggests that primary metabolism and other basic cellular functions could be rewired in the resistant species to limit infection. Transcriptomes of young flushes of the three species are very different, supporting the existence of distinct biochemical niches for the bacterium. These findings suggest that both intrinsic metabolic inadequacies to *C*Las survival, as well as inducible reprogramming of physiological functions upon *C*Las recognition, could orchestrate together restriction of bacterial multiplication in these resistant hosts.

## Introduction

During the last decade, compelling models of plant–pathogen interactions have been established, mostly based on molecular studies of mesophyll cell-infecting pathogens (Dodds and Rathjen, 2010; Asai and Shirasu, 2015; Ceulemans et al., 2021). Local immune activation involves the activity of two classes of receptors: plasma membrane-localized receptors that typically recognize conserved microbial patterns in the apoplast, named as pathogen-associated-molecular-patterns (PAMPs). PAMPs detection constitutes the first tier of active plant immunity, which involves an influx of extracellular Ca_2_^+^ into the cytosol, an oxidative burst, and a massive reprogramming of the transcriptome in the first hours after PAMP recognition. Some pathogens overcome this PAMP-triggered immunity (PTI) by secreting effectors that manipulate cellular processes and suppress PTI. In resistant plants, targeted recognition of these effectors by cytoplasmic receptors forms the second tier of active plant immunity, called effector-triggered immunity (ETI). Both responses are triggered very quickly, ensuring, for many interactions, the complete deployment of defenses withing the first days after pathogen detection. The ability of a pathogen to overcome these two defensive lines within this time frame determines its success in establishing the infection (Thomma et al., 2011; Bigeard et al., 2015; Remick et al., 2023).

Some of the most devastating plant pathogens, however, do not infect mesophyll cells. They are delivered into the sieve cells of the phloem by feeding insects using specialized mouth parts called stylets, which pierce the plant tissue and directly access the nutrient-rich phloem vessels. The stylets travel through the apoplast to reach the phloem, secreting saliva to form a hard sheath that seals off plant cell leaks caused by the penetration process, causing limited wounding to the plant. Thus, those pathogens are directly delivered to the sieve cell cytoplasm, which lacks a nucleus, and its potential to mount an effective immune response is uncertain (Jiang et al., 2019; Huang et al., 2020). In addition, the high genome reduction rate of these pathogens, which makes them very dependent on host metabolism for survival, implies a lack of typical transmembrane systems for secretion of effectors (Duan et al., 2009; Oshima et al., 2013; Huang et al., 2020; Rattner et al., 2021). Under this scenario, which aspects of the aforementioned plant immunity are applicable to these interactions remain unknown.

One of the most ravaging diseases caused by phloem-inhabiting prokaryotic pathogens is Huanglongbing (HLB), also known as citrus greening. Since 2004 and 2005, when it was respectively detected in São Paulo (Brazil) and in Florida (USA), two of the most significant citrus-growing regions worldwide, it has caused billions of dollars in losses. In Brazil, despite successful management strategies adopted in the last years, the HLB incidence in commercial groves continues to increase, now affecting more than 44% of sweet orange trees in the citrus belt of São Paulo and Minas Gerais states (Fundecitrus, 2024). In Florida, the citrus industry relies on the limited production of more than 99% of infected trees (Li et al., 2020b). The disease has spread to almost all citrus-producing regions worldwide, except Australia and Mediterranean Basin.

The predominant HLB pathogen is the non-culturable, gram-negative α-proteobacterium ‘*Candidatus* Liberibacter asiaticus’ (*C*Las), which is mainly vectored by the Asian citrus psyllid (ACP) *Diaphorina citri* (Kuwayama) (Sternorrhyncha: Psyllidae) (Burckardt et al., 2021). Foliar symptoms include asymmetrical chlorosis known as blotchy mottle, enlarged veins, and intense canopy defoliation (Bové, 2006), caused by disturbance of the transport system between source and sinks, with starch accumulation in plastids, chloroplast disruption, and plugging of sieve cells via callose deposition (Bové, 2006; Andrade et al., 2019; Pandey and Wang, 2019). In field trees, these symptoms appear within some months to years after infection, depending on the tree’s age and size (Gottwald, 2010; Lopes et al., 2009). During the asymptomatic phase, infected plants remain indistinguishable from *C*Las-free plants and are an important source for the disease to spread within the grove, which complicates disease management (Lopes et al., 2009; Lee et al., 2015). How *C*Las can evade immunity and remain undetected by its host for such a long period is still unknown. *C*Las does not possess the conserved secretion mechanism to deliver effectors into plant cells, although most components needed to generate a functional Sec-dependent secretion system are present in its genome (Duan et al., 2009). The Sec-dependent secretion system has been hypothesized to be essential for pathogens like *C*Las or phytoplasmas to infect plants (Sugio et al., 2011; Yan et al., 2013) and it has been reported that *C*Las genes belonging putatively to this Sec-dependent system are upregulated in planta, but not in *C*Las-infected psyllids (Yan et al., 2013).

Although sensitivity to *C*Las varies among *Citrus* species and varieties, no complete resistance has been documented in this genus (Folimonova et al., 2009). However, Oceanian *Citrus* species formerly included by Swingle and Reece (1967) within the *Microcitrus* and *Eremocitrus* genera have been reported to be resistant to *C*Las (Ramadugu et al., 2016; Alves et al., 2021b, Alves et al., 2022). Moreover, partial or complete resistance has been reported in certain related genera within the family Rutaceae subfamily Aurantioideae (Ramadugu et al., 2016; Beloti et al., 2018; Cifuentes-Arenas et al., 2019; Alves et al., 2021a, Alves et al., 2021b, Alves et al., 2022). The monoembryonic orange jasmine (*Murraya paniculata* (L.) Jack) and curry leaf (*Bergera koenigii* L.), are good hosts for ACP (Damsteegt et al., 2010; Teck et al., 2011; Westbrook et al., 2011), being even more attractive to ACP females than sweet orange (Beloti et al., 2017; Tomaseto et al., 2019; Eduardo et al., 2022). *M. paniculata* is considered a transient host for *C*Las while *B. koenigii* is *C*Las-immune (Damsteegt et al., 2010; Beloti et al., 2018; Cifuentes-Arenas et al., 2019), providing a unique opportunity to explore distinct molecular mechanisms potentially involved in HLB-resistance. Recently, in a simulation of the natural entry of *C*Las in the plant host, Alves et al. (2021a) studied *C*Las dynamics in sweet orange *Citrus × sinensis* (L.) Osbeck ‘Valencia’, in *M. paniculata*, and in *B. koenigii* in the first stages of these plant-bacteria interactions. Using a well-controlled challenge-inoculation system with infectious psyllids, critical time points for these plant species with different responses to *C*Las infection were identified. In all three experimental systems, *C*Las was successfully released into the phloem by ACP, and bacterial titers decreased during the first days after inoculation. Subsequent increases in bacterial titers were observed for each host: while an exponential increase occurred in *C. × sinensis* up to the 40^th^ day post-inoculation (DAI), from which a plateau was reached, *C*Las failed to successfully replicate in *B. koenigii*. In *M. paniculata*, *C*Las titers were generally lower than in *C. × sinensis*, and titer dynamics resembled that of *C. × sinensis* up to the 30^th^ DAI, from which bacterial titers progressively decreased to undetectable levels (Alves et al., 2021a). These observations suggest that the first weeks after bacterial release into the phloem by ACP are critical for the establishment of the bacterium in each host, providing an excellent system to investigate molecular mechanisms leading to either resistance or susceptibility.

Here, a thorough transcriptomic analysis of new flushes of *C. × sinensis, B. koenigii* and *M. paniculata* was performed to study host gene expression dynamics after the ACP-vectored transmission of *C*Las. Our results suggest that the different responses to the bacterium in the three species are not due to the deployment of classical immunity in the resistant species pointing to a mechanism in which primary metabolism and other basic cellular functions are rewired to limit bacterial infection.

## Materials and methods

### Plants and growth conditions

Rearing of insects and plant maintenance were carried out in a controlled environmental room with 55% and 78% relative humidity, and daily temperature of 24^°^C-27^°^C. Lights were 20-30 cm above the plants with a photoperiod of 12 h of light (300 µmol m^-2^ s^-1^) and 12 h of darkness. Plants were grown in 300 mL conical tubes (6.5 x 5.9 cm, upper x lower diameter; 16 cm, height), filled with coconut fiber. In total, 51 two-year-old plants of *Citrus × sinensis* (L.) Osbeck ‘Valencia’ grafted on *C. × limonia* Osbeck (‘Rangpur’ lime), 39 two-year-old seedlings of *M. paniculata* and 39 of *B. koenigii* were used.

### Rearing of *C*Las-infected and *C*Las-negative psyllids

Insect rearing and inoculation followed the methodology described by Lopes and Cifuentes-Arenas (2021). *C*Las-positive (real-time polymerase chain reactions (qPCR) Ct average of 21.82 ± 0.79) ‘Valencia’ sweet oranges grafted on ‘Swingle’ citrumelo (*C. × paradisi* Macfad. *× Poncirus trifoliata* L. Raf.) were used for obtaining *C*Las-positive insects. Those *C*Las-infected plants were pruned and when new flushes reached the V2 stage (Cifuentes-Arenas et al., 2018), four 10 to 20-days-old *C*Las-negative psyllids were confined per shoot. After 7 days of oviposition, adults were removed, and the eggs developed into nymphs and hatched into adults (named F1). When these F1 adults reached ~5 days, *C*Las presence was assessed by qPCR in a random sampling of three insects per plant. The remaining insects were kept confined in the *C*Las-positive plants until they were 15 to 20 days-old, and used for challenge inoculation experiments. *C*Las-negative insects were obtained using the same methodology on qPCR-negative plants. Adult insects used for oviposition were obtained from a colony kept at Fundecitrus, in which insects are continuously reared on healthy *M. paniculata* seedlings (Skelley and Hoy, 2004).

### Psyllid challenge inoculation

*C. × sinensis* ‘Valencia’ plants, *M. paniculata* and *B. koenigii* seedlings were pruned at 15-25 cm to promote new flushes to sprout. A V2 flush was selected, and the remaining ones were eliminated. Five 15-20day-old *C*Las-positive or negative adult psyllids were confined to a single flush per plant for 48 h (inoculation access period, IAP). Twenty-four ‘Valencia’ sweet orange plants and 18 of each *M. paniculata* or *B. koenigii* seedlings were exposed to *C*Las-positive psyllids and the same number to *C*Las-negative ones. After the IAP, adults were removed and stored at -20°C. Fifteen days after psyllid removal, all plants were sprayed with insecticide (Abamectin EC, 7.2 g of active ingredient per 1 L of water) to eliminate any eggs or nymphs present.

### Plant sampling procedures

Three plants per species were randomly selected as controls prior to the confinement with the insect (called here as “prior”). Samples were randomly collected at 0 (t0, day of psyllid removal), 10 (t10), 20 (t20), 30 (t30), 60 (t60),120 (t120) and 180 (t180) days after psyllid removal. The tissue that was in contact with the psyllid was frozen in liquid nitrogen and stored at -80°C. Due to shoot’s growth over time, the total collected tissue varied between samples. Each plant was used at a single time point, with 5-8 plants being used per time point for each species.

### Evaluation of *C*Las multiplication

Total DNA was extracted from 0.1 g of frozen tissue. Psyllid DNA was extracted as described by Murray and Thompson (1980), with modifications by Alves et al. (2021a). After DNA precipitation with 0.6 V of isopropanol, DNA was washed twice with 70% ethanol and resuspended in 50 uL of Milli-Q^®^ water. *C*Las presence was assayed by qPCR using *C*Las 16S rRNA gene sequence, using 100 ng of total DNA, TaqMan^®^ PCR Master Mix (1x) (Invitrogen, Carlsbad, CA, United States), and HLBas primer-probe (0.5 µM/0.2 µM) in a StepOnePlus thermocycler (Applied Biosystems, California, USA) (Li et al, 2006). The mitochondrial gene cytochrome oxidase (COX) and a psyllid wingless (wg) gene region were used as internal controls to assess plants and psyllid DNA quality, respectively (Li et al., 2006; Manjunath et al., 2008). For *C*Las quantification, the linear relationship between the cycle threshold (Ct) and the 16S rRNA log was used (Lopes et al., 2013). Samples were considered *C*Las-positive when the Ct was lower than 34.0.

### RNA extraction and sequencing

Samples for transcriptomic analysis were taken before the exposure to psyllids (prior), just after removal of the psyllids (t0) and after 10 (t10), 20 (t20), 30 (t30) and 60 (t60) days. Three individual flushes per species and time-point, which were exposed to either *C*Las-positive or negative psyllids, were selected. Each one was considered a biological replicate. Additionally, for time-point t10, three more replicates per species were used. Total RNA was extracted using the RNeasy Mini Kit (Qiagen, Valencia, CA, USA).

Total RNA concentration was calculated by Quant-IT RiboGreen (Invitrogen, #R11490). To assess the integrity of the total RNA, samples are run on the TapeStation RNA screentape (Agilent, #5067-5576). Only high-quality RNA preparations, with RIN greater than 7.0, were used for RNA library construction. A library was independently prepared with 0.5ug of total RNA for each sample by Illumina TruSeq Stranded Total RNA Library Prep Plant Kit (Illumina, Inc., San Diego, CA, USA, # 20020611). The first step involves removing the rRNA in the total RNA. Following this step, the remaining mRNA is fragmented into small pieces using divalent cations under elevated temperature. The cleaved RNA fragments are copied into first strand cDNA using SuperScript II reverse transcriptase (Invitrogen, #18064014) and random primers. Second strand cDNA synthesis was performed using DNA Polymerase I, RNase H and dUTP. These cDNA fragments were repaired by addition of a single ‘A’ base, and ligation of the adapters. The products are purified and enriched with PCR to create the final cDNA library. The libraries were quantified using KAPA Library Quantification kits for Illumina Sequencing platforms according to the qPCR Quantification Protocol Guide (KAPA BIOSYSTEMS, #KK4854) and qualified using the TapeStation D1000 ScreenTape (Agilent Technologies, # 5067-5582). Indexed libraries were then submitted to an Illumina NovaSeq (Illumina, Inc., San Diego, CA, USA), and the paired-end (2×150) sequencing was performed by the Macrogen Incorporated.

### *Bergera koenigii* transcriptome assembly

*B. koenigii* transcriptome was assembled with Trinity v2.15.2 (Grabherr et al., 2011) using default parameters. Transcripts were collapsed into SuperTranscripts using the corresponding script from the Trinity package. Analysis of gene space completeness was performed with BUSCO “transcripts” mode (Manni et al., 2021) and the Viridiplantae Odb10 dataset. Presence of contaminants was assessed using Blobtools, version 1.1.1 (Challis et al., 2020). Reads were remapped against the created supertranscripts using the BWA (Burrows-Wheeler Aligner) program v.0.7.17-r1188 (Li and Durbin, 2009). Then, bam files were merged and indexed using Samtools (Li et al., 2009). Finally, Diamond Blastx against Uniprot Trembl database was run (Buchfink et al., 2015) and a Blobtools database was created. Results were filtered by removing sequences not associated with the Streptophyta Tag.

### Transcriptome analysis

Adapter removal and quality trimming of raw reads was done with Cutadapt v3.7 (Martin, 2011). Clean reads were analyzed using FastQC v0.11.9 (Andrews, 2010). Clean read pairs longer than 20 nt were mapped to the combined genome of *Candidatus* Liberibacter asiaticus (https://www.ncbi.nlm.nih.gov/nuccore/NZ_CP019958) with either the *C. × sinensis* SWO.v3.0 (http://citrus.hzau.edu.cn/download.php), or the *M. paniculata* genome assembly (provided by Dr. Concetta Licciardello, Council for Agricultural Research and Economics, CREA), or the *Bergera koenigii de novo* transcriptome assembly (this study, see above) using STAR (Dobin et al., 2013). Number of reads mapped to one and only one of the annotated genes of the genome (uniquely mapped) was also obtained with STAR. In total, more than 855 million uniquely-mapped gene-assigned reads were obtained for *C. × sinensis*, 742 million for *M. paniculata* and 1376 million for *B. koenigii* (**Supplementary Table 1**). Differential expression analysis was performed with DESeq2 (Love et al., 2014). Principal component analysis was done on the NetworkAnalyst 3.0 suite (Zhou et al., 2019). Gene Set Enrichment Analysis (GSEA) (Subramanian et al., 2005) was used for pairwise comparisons within a species and Over-Representation Analysis (ORA) (Boyle et al., 2004) for pairwise comparisons among orthologous groups. GO categories were summarized by removing redundant terms according to ReviGO (Supek et al., 2011). Categories with frequency < 5 were selected and ranked by uniqueness. Bubble and UpSet plots were done using SRplot (Tang et al., 2023).

## Results

### CLas establishment and shoot ontogeny differs between *C. × sinensis*, *M. paniculata* and *B. koenigii*

**Figure 1:**
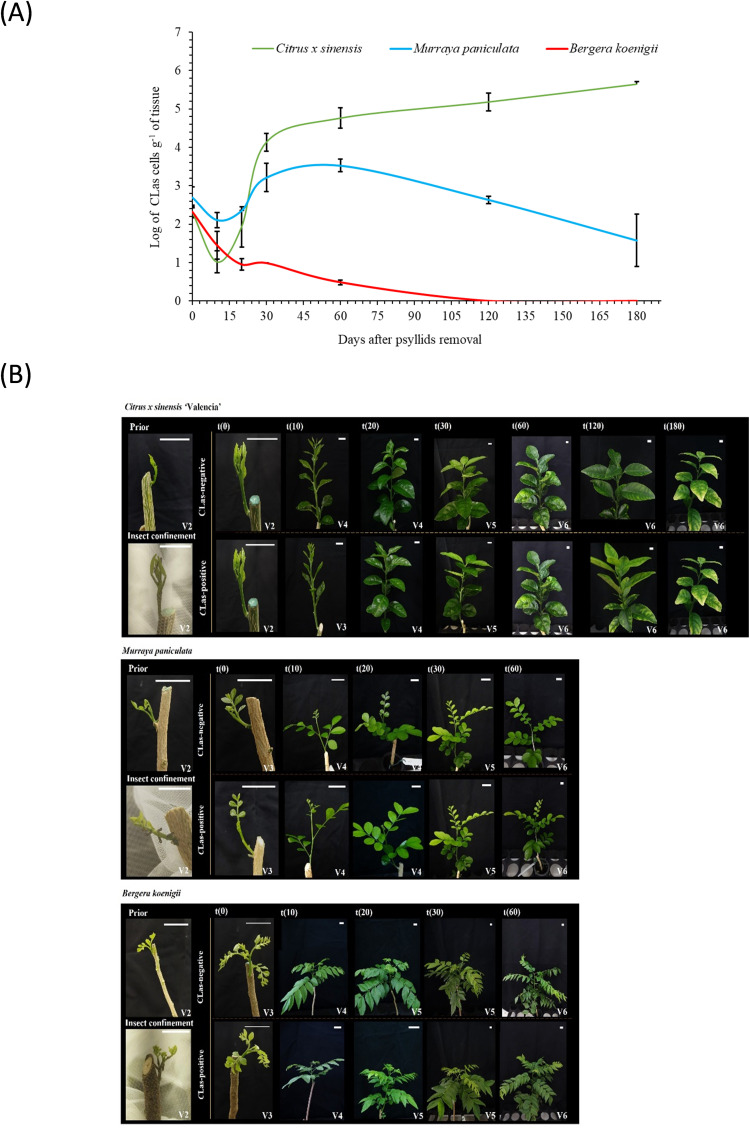
Shoot ontogeny and ‘*Candidatus* Liberibacter asiaticus’ population dynamics. **(A)** Evolution of the ‘*Candidatus* Liberibacter asiaticus’ population dynamics over time in new shoots of *Citrus × sinensis, Murraya paniculata*, and *Bergera koenigii*, following a 48 h period of *C*Las-exposed *Diaphorina citri* confinement. **(B)** Representative aspect of shoots from *Citrus × sinensis*, (top panels) *Murraya paniculata*, (middle panels) and *Bergera koenigii* (bottom panels) prior to insect confinement, at the confinement day, 48 h after exposure to *C*Las-positive or *C*Las-negative *Diaphorina citri* (t0) and 10 (t10), 20 (t20), 30 (t30), 60 (t60), 120 (t120) and 180 (t180) days after insect removing. White bar length in each photo corresponds to 2 cm.

After the 48h IAP (t0), psyllid survival rate (avg. 98.5%) and *C*Las titer in psyllids (avg. 5.1 ± 0.15 log cells/g of tissue) were similar regardless of the plant species (**Supplementary Table 2**). Similarly, the amount of initial bacterial load released in the plant vasculature after IAP did not differ between species, with averages of 2.42 ± 0.12 log cells/g of tissue, which is close to the limit of detection of the qPCR technique employed (**Figure 1A**; **Supplementary Table 3**). This was followed by a steep decrease up to t20. Subsequently, in *C. × sinensis*, *C*Las titer increased exponentially, until t30, increasing ~1 log until t60. In *M. paniculata*, the initial decrease was gradual, as well as the subsequent exponential increase until t30. Maximum titer was observed at t60, slowly decreasing thereafter. In *B. koenigii*, a drastic reduction in *C*Las titer following psyllid removal was observed at t10, and the subsequent increase noticed for the other two species from t20 onwards was not observed (**Figure 1A**).

Flush ontogeny along the experiment was determined according to Cifuentes-Arenas et al. (2018). The three species followed different ontogeny patterns, and the presence of the bacterium affected their development differently. *C. × sinensis* V2 flushes exposed to *C*Las-negative psyllids entered the maturation phase (V4) at t10 with leaves fully expanded at t30 (V5), and dormancy stage (V6) from t60 onwards. Flushes exposed to *C*Las-infected psyllids remained in developmental phase (V3) at t10, reached maturation phase (V4) at t20 and then followed the same dynamics as *C*Las-negative flushes. For *M. paniculata* and *B. koenigii*, developmental patterns were different to those of *C. × sinensis*. In both species and for either *C*Las-negative or positive flushes, initial V2 flushes entered the developmental phase (V4) at t10 with fully-expanded leaves at t20 (V5), earlier than in *C. × sinensis* (**Figure 1B**).

### Transcriptomes of *C. × sinensis*, *M. paniculata* and *B. koenigii* are dramatically reprogrammed during flush growth

A thorough transcriptomic experiment was designed to evaluate the response to *C*Las in the three species. *C. × sinensis* and *M. paniculata* clean reads were mapped against the *C. × sinensis* SWO.v3.0 genome or the *M. paniculata* genome. Cross-mapping of *B. koenigii* clean reads against these two genomes revealed mapping percentages of 55% and 43%, respectively. Therefore, a *de novo B. koenigii* transcriptome was assembled using the whole set of reads obtained in this study. This assembly yielded a total of 25200 supercontigs, with a genome completeness of 89%. This *de novo* transcriptome was used to map *B. koenigii* reads.

**Figure 2:**
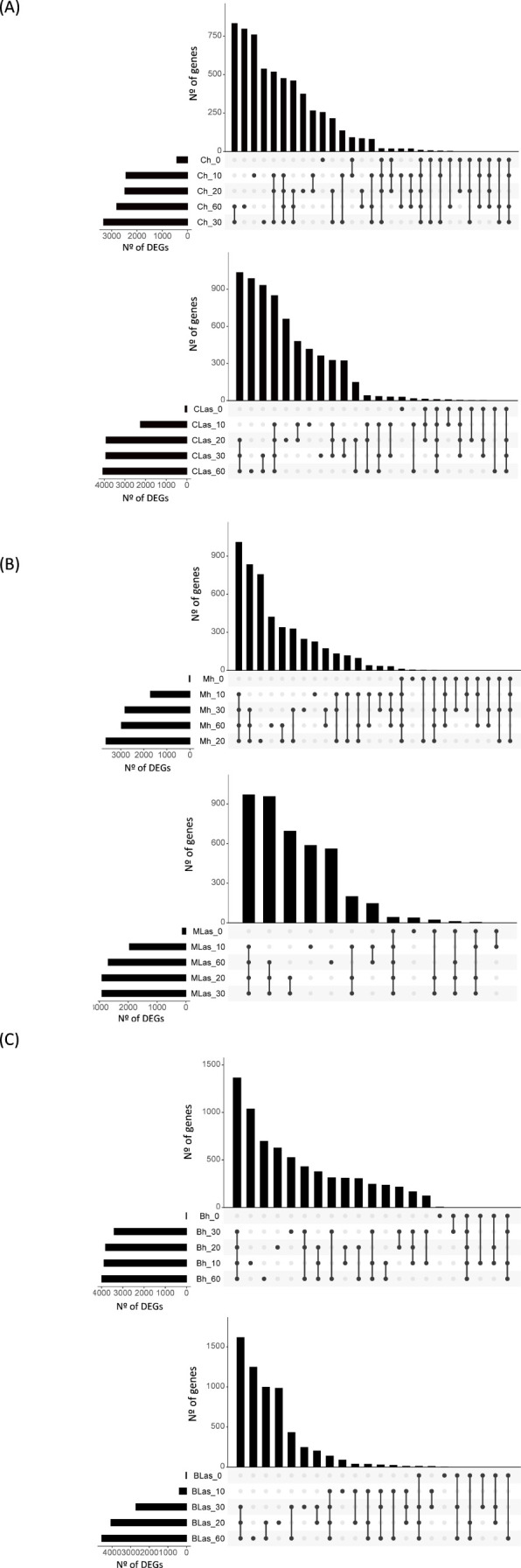
Transcriptome changes in *Citrus* ×*sinensis, Murraya paniculata* and *Bergera koenigii* flushes along the first 60 days after exposure to psyllids. **(A)** Number of up-regulated genes in *Citrus × sinensis* samples taken immediately (CLas_0, Ch_0), and after 10 (CLas_10, Ch_10), 20 (CLas_20, Ch_20), 30 (CLas_30, Ch_30) or 60 (CLas_60, Ch_60) days after exposure to *C*Las-negative (Ch, top) or *C*Las-positive (CLas, bottom) psyllids when compared against flushes before the exposure to psyllids (prior). **(B)** Number of up-regulated genes in *Murraya paniculata* samples taken immediately (MLas_0, Mh_0), and after 10 (MLas_10, Mh_10), 20 (MLas_20, Mh_20), 30 (MLas_30, Mh_30) or 60 (MLas_60, Mh_60) days after exposure to *C*Las-negative (Mh, top) or *C*Las-positive (MLas, bottom) psyllids when compared against flushes before the exposure to psyllids (prior). **(C)** Number of up-regulated genes in *Bergera koenigii* samples taken immediately (BLas_0, Bh_0), and after 10 (BLas_10, Bh_10), 20 (BLas_20, Bh_20), 30 (BLas_30, Bh_30) or 60 (BLas_60, Bh_60) days after exposure to CLas-negative (Bh, top) or CLas-positive (BLas, bottom) psyllids when compared against flushes before the exposure to psyllids (prior). *C*Las: *Candidatus* Liberibacter asiaticus. DEGs: Differentially expressed genes. In each graph, total number of DEGs (X axis) at each time point (Y axis) is shown in bottom-left panels. Intersection of sets of genes at multiple time points is shown in top right panels. Each column corresponds to a time point or set of time points (dots connected by lines below the X axis) containing the same DEGs. The time points shared are indicated in the graphic below the column, with the time points on the left.

First, transcriptome changes occurring in flushes along the first 60 days after exposure to *C*Las-negative psyllids were analyzed. Overall, gene expression was dramatically altered as compared to that of flushes prior to the inoculation period in the three species (**Figures 2A–C**; **Supplementary Figure 1**). Massive transcriptome changes were first observed at t10, with thousands of differentially expressed genes (DEGs), and a substantial number of changes also found at later time points, when flushes were at V5 or V6 stages. Similarly, flushes exposed to *C*Las-positive psyllids also exhibited massive gene-expression changes at t10, except for flushes of *B. koenigii* exposed to *C*Las-positive psyllids, whose transcriptional reprogramming was mainly triggered at t20 (**Supplementary Table 4**). These results suggest that, in general, gene expression is highly mobilized upon flush growth in shoots exposed to ACP, regardless of whether they were exposed to *C*Las-negative or positive psyllids.

### *C. × sinensis* flush transcriptome is not dramatically altered upon *C*Las infection up to 60 days after inoculation

**Figure 3:**
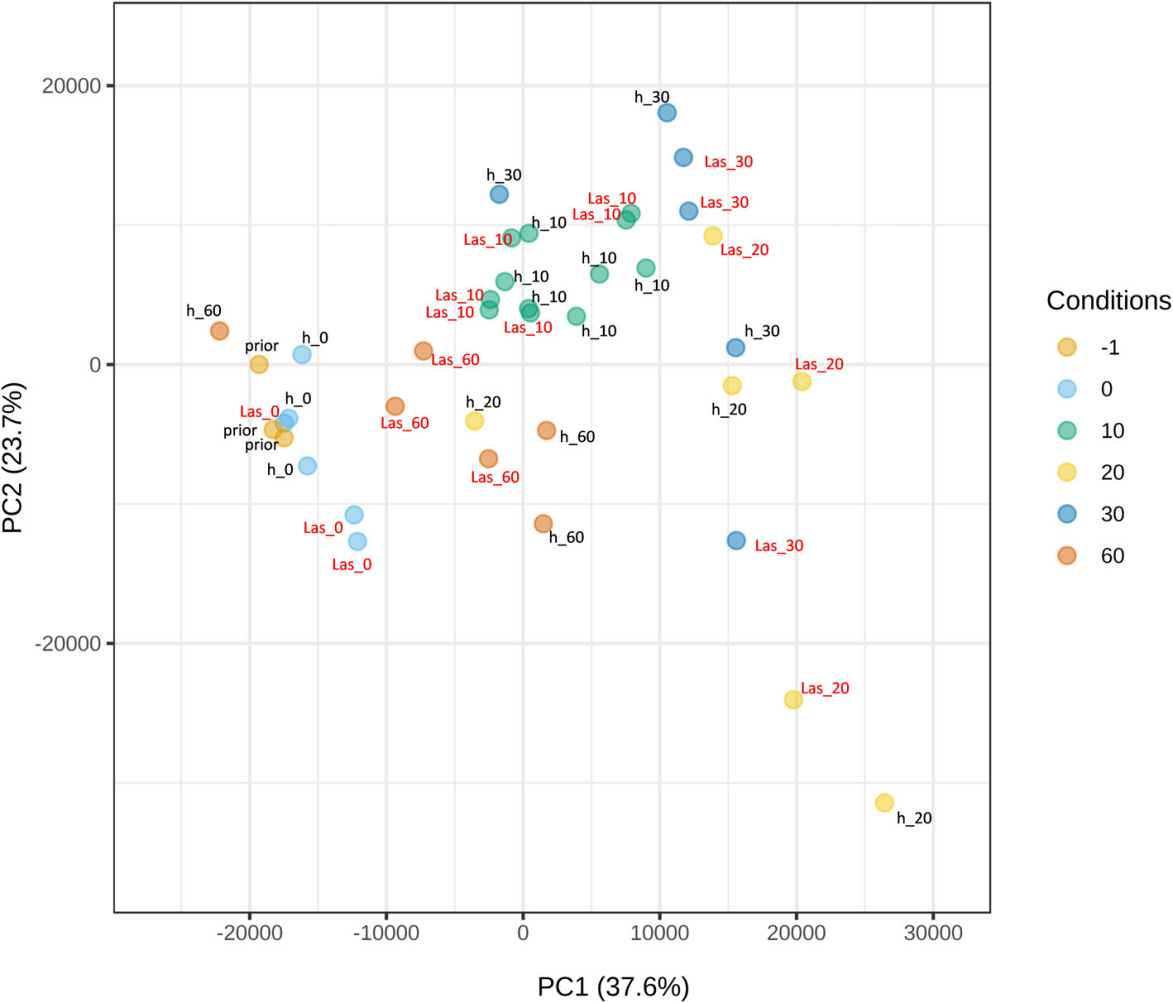
Principal Component Analysis (PCA) plot of *Citrus* × *sinensis* samples. Samples were taken before the exposure to psyllids (prior), immediately after exposure (0) or 10, 20, 30 or 60 days after exposure. h: samples exposed to *C*Las-negative psyllids; Las: samples exposed to *C*Las-positive psyllids. PC, principal component.

To identify differentially expressed genes (DEGs) in *C. × sinensis* upon *C*Las inoculation, paired-wise comparisons were performed between flushes exposed to *C*Las-negative or *C*Las-positive psyllids at every time-point. At t0, 167 genes were found differentially expressed after exposure to the bacterium, 123 genes at t10, 8 genes at t30 and 48 genes at t60 (**Supplementary Table 5**). This low number of differences was also reflected in PCA biplots shown in **Figure 3**, in which samples were roughly clustered according to flush developmental stage but not to exposure to *C*Las. Despite the low number of DEGs, some gene ontology categories were found enriched after gene-set-enrichment-analysis (GSEA) for every paired-wise comparison (**Supplementary Table 6**). A reduction of metabolic activity and growth-related processes in t0 flushes exposed to *C*Las was observed. Other enriched categories include an increase in DNA damage response in t10, in cell wall remodeling pathways in t30, and a decrease in translation and energy processes at t60. Our data suggest that the presence of the bacterium in *C. × sinensis* flushes did not cause massive transcriptional changes up to 60 days after inoculation.

### Flush transcriptome is not dramatically altered upon *C*Las infection in *M. paniculata* or *B. koenigii* along 60 days after inoculation

**Figure 4:**
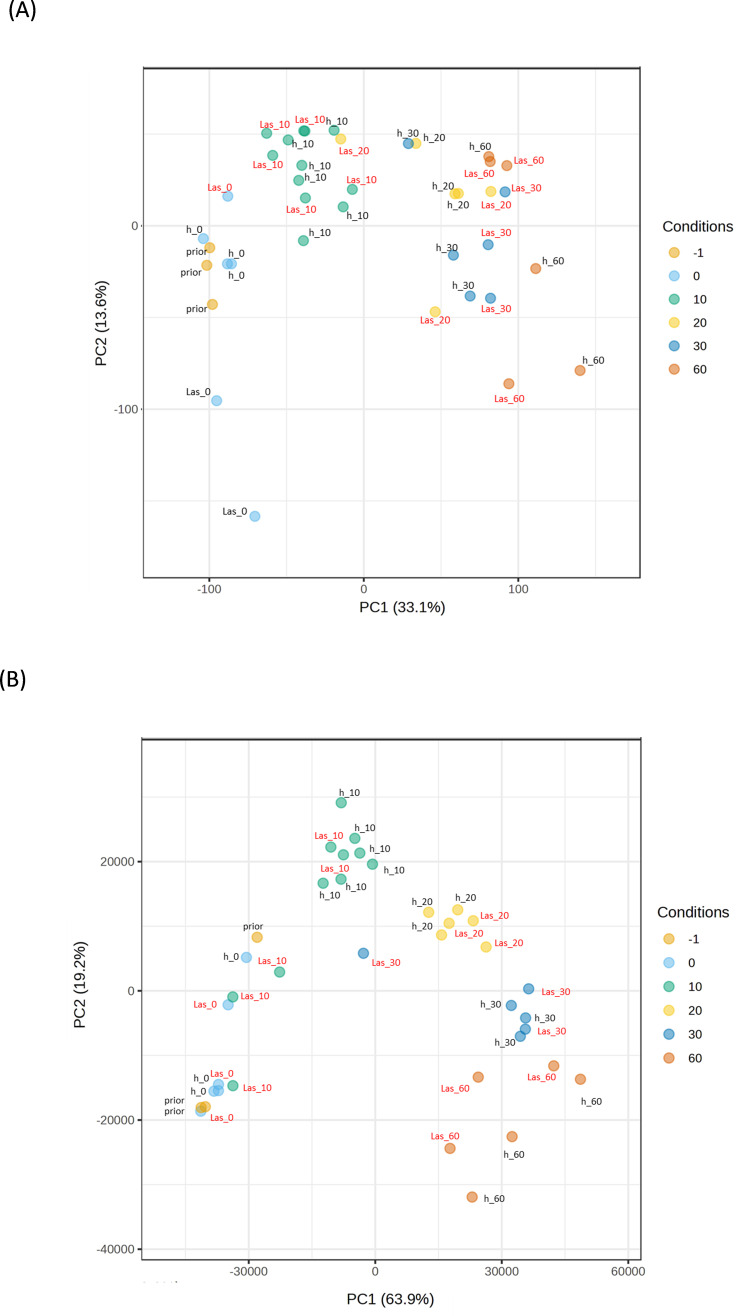
Principal Component Analysis (PCA) plot of *Murraya paniculata* and *Bergera koenigii* samples. **(A)** PCA plot for *Murraya paniculata* samples. **(B)** PCA plot for *Bergera koenigii* samples. Samples were taken before the exposure to psyllids (prior), immediately after exposure (0) or 10, 20, 30 or 60 days after exposure. h: samples exposed to *C*Las-negative psyllids; Las: samples exposed to *C*Las-positive psyllids. PC, principal component.

**Figure 5:**
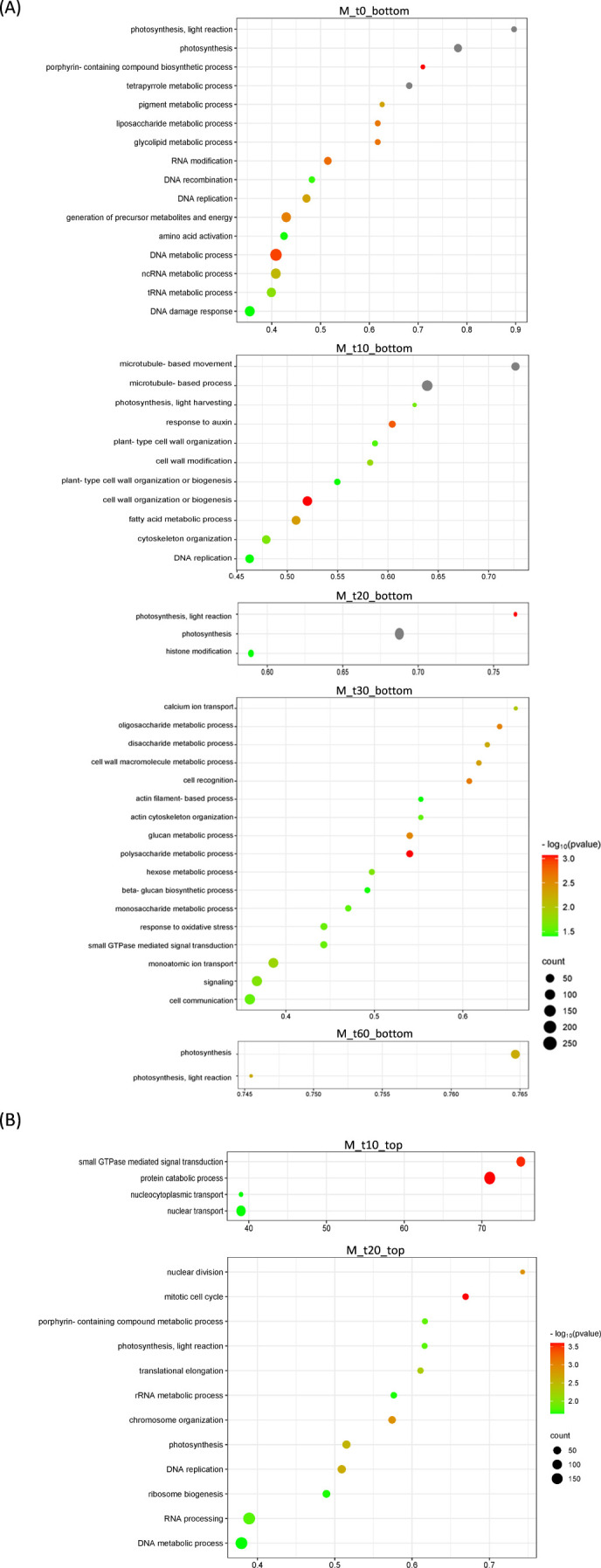
Bubble plots showing Gene Ontology (GO) terms enriched in *Murraya paniculata* differentially expressed genes. **(A)** Top-20 nonredundant enriched GO terms after Gene Set Enrichment Analysis in the bottom-ranked genes (bottom) after Deseq2 statistical test. These represent genes less expressed in *C*Las-infected flushes at different time points after psyllid exposure (M_t0 to M_t60). **(B)** Top-20 nonredundant enriched GO terms after Gene Set Enrichment Analysis in the top-ranked genes (top) after Deseq2 statistical test. These represent genes more expressed in *C*Las-infected flushes at different time points after psyllid exposure (M_t10 and M_t20). GO terms for the remaining time points are detailed in **Supplementary Table 7**. GO terms were obtained using Panther and filtered using ReviGO to remove semantically redundant terms. GO terms with frequency<10% were selected and ranked by dispensability. Gene ratio: Ratio of upregulated genes in a given category divided by total number of genes in this category. Counts, number of upregulated genes in a given category. −log P (P-value in log scale after false discovery rate correction).

**Figure 6:**
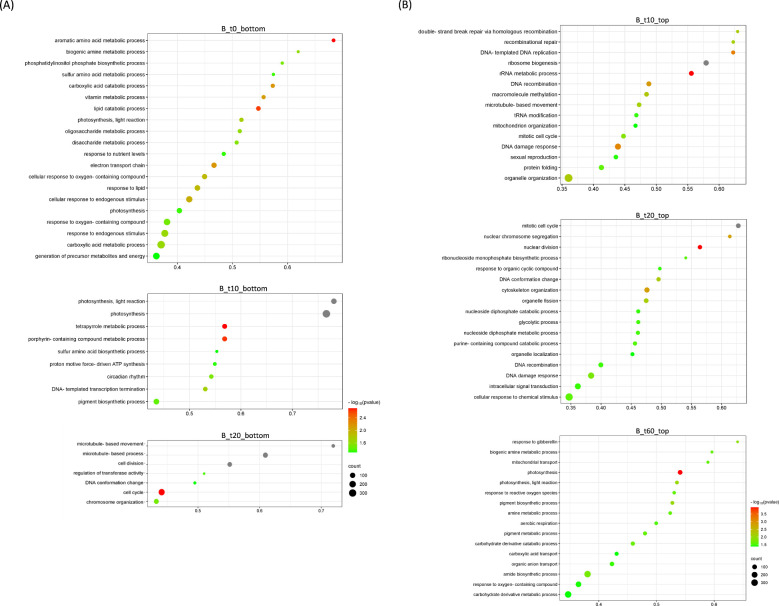
Bubble plots showing Gene Ontology (GO) terms enriched in *Bergera koenigii* differentially expressed genes. **(A)** Top-20 nonredundant enriched GO terms after Gene Set Enrichment Analysis in the bottom-ranked genes (bottom) after Deseq2 statistical test. These represent genes less expressed in *C*Las-infected flushes at different time points after psyllid exposure (B_t0, B_t10; B_t20). **(B)** Top-20 nonredundant enriched GO terms after Gene Set Enrichment Analysis in the top-ranked genes (top) after Deseq2 statistical test. These represent genes more expressed in *C*Las-infected flushes at different time points after psyllid exposure (B_t10, B_t20; B_t60). Number of GO terms for the remaining time points are detailed in **Supplementary Table 8**. GO terms were obtained using Panther and filtered using ReviGO to remove semantically redundant terms. GO terms with frequency<10% were selected and ranked by dispensability. Gene ratio: Ratio of genes in a given category divided by total number of genes in this category. Counts, number of genes in a given category. −log P (P-value in log scale after false discovery rate correction).

A parallel analysis was performed for the transient host *M. paniculata* and the fully resistant *B. koenigii*. In both species, paired-wise comparison again indicated that only a few significant changes were occurring between flushes that were exposed to *C*Las-negative psyllids and those that were exposed to *C*Las-positive ones, with less than 100 DEGs per time-point from t10 to t30 (**Supplementary Tables 7**, **S8**). In *M. paniculata*, 17 genes were up-regulated and only 5 down-regulated at t0 after inoculation with *C*Las-positive psyllids, 4 and 23 genes at t10, 14 and 79 at t20, 24 and 70 at t30 and 41 and 90 at t60 (up- and down-regulated, respectively). In *B. koenigii*, 28 genes were up-regulated and 85 genes down-regulated after inoculation with *C*Las-positive psyllids, 19 and 4 genes at t10, 14 and 34 at t20, 16 and 12 at t30, and 560 and 117 at t60 (up- and down-regulated, respectively). PCA plots revealed that samples were clustered according to flush developmental stage and not to the exposure or not to *C*Las (**Figures 4A, B**). In *B. koenigii*, a closer view to the PCA plot revealed that, for *C*Las-positive challenge, three out of the six replicates in t10 had a transcriptional signature that was closer to samples at t0, whereas in two of them it was closer to t10 samples exposed to *C*Las-negative psyllids. The remaining replicate displayed a signature unrelated to the rest of the samples and is not plotted (**Figure 4B**). This observation suggests the existence in this time point of samples of different nature among those exposed to *C*Las. GSEA analysis identified enriched categories which give insight into the transcriptional alterations taking place in *C*Las-infected flushes. Among others, it brought our attention the repression of photosynthesis-related processes when *C*Las was present, observed at t0, t10, t30 and t60 for *M. paniculata*, and t0 and t10 for *B. koenigii*, or the downregulation of pathways related to sulfur amino acids in t0 and t10 in *B. koenigii* (**Figures 5**, **6**). The complete list of categories is shown in **Supplementary Tables 9**, **10**. No categories related to plant immunity or defense responses to bacteria were detected in any time point in any of the two species., In summary, *C*Las presence on *M. paniculata* or *B. koenigii* flushes seems not to cause massive changes in gene expression up to 60 days post-inoculation, with some biochemical reprogramming events taking place.

### Transcriptomes of young flushes reveal distinct biochemical potentials for the three species that could explain differences in their response to *C*Las

The previous results suggest that *M. paniculata* and *B. koenigii* are not deploying a canonical immune response after *C*Las inoculation. It also suggests that gene expression changes upon *C*Las detection in flushes in both species are associated to biochemical rearrangements. To assess whether survival and multiplication of *C*Las in the different hosts is also influenced by the biochemical potential of the tissues in which *C*Las was initially inoculated by ACP, flush transcriptomes before bacterial inoculation (prior) were compared.

**Figure 7:**
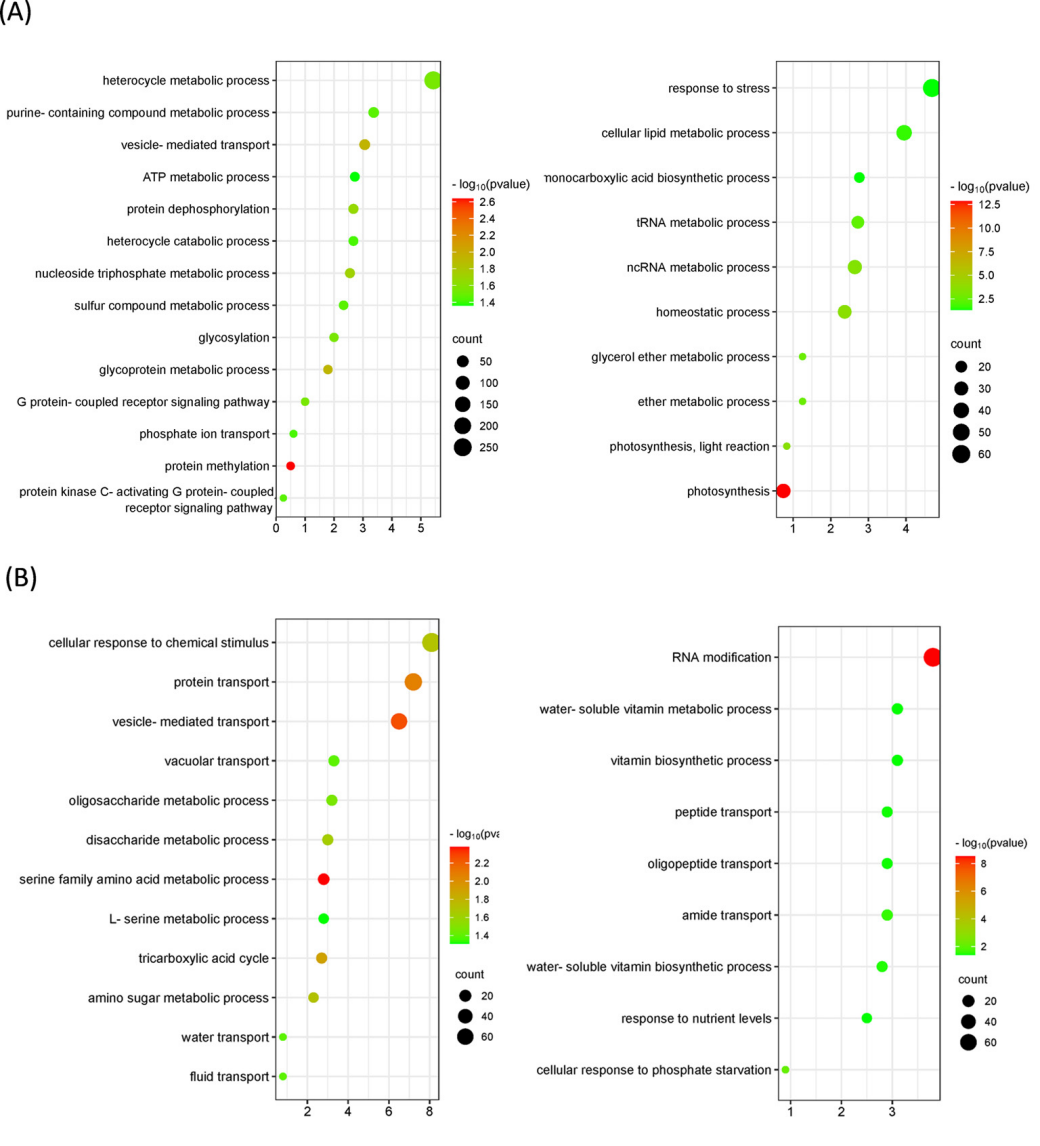
Bubble plots showing Gene Ontology (GO) terms enriched in orthologous groups between *Citrus* × *sinensis* and *Murraya paniculata* or *Bergera koenigii* flushes before psyllid exposure. **(A)** Top-20 nonredundant enriched GO terms among orthologous genes more (left) or less (right) expressed in *Citrus × sinensis* as compared to *Murraya paniculata*. **(B)** Top-20 nonredundant enriched GO terms among orthologous genes more (left) or less (right) expressed in *Citrus × sinensis* as compared to *Bergera koenigii*. GO terms were obtained using Panther and filtered using ReviGO to remove semantically redundant terms. GO terms with frequency<5% were selected and ranked by dispensability. Gene ratio, Ratio of genes in a given category divided by total number of genes in this category. Counts, number of genes in a given category. −log P (P-value in log scale after false discovery rate correction).

Gene expression was analyzed for groups of orthologous genes. Nearly 14000 and 9000 orthologous groups were identified between *C. × sinensis*, *M. paniculata* and *B. koenigii*, respectively. Many of those genes were found differentially expressed in non-inoculated flushes of the three species. More than 2500 genes were found more expressed in *B. koenigii* than in *C. × sinensis* flushes, and more than 2000 were less expressed. Similarly, more than 2600 genes were found more expressed in *M. paniculata* than in *C. × sinensis*, whereas more than 2900 genes were less expressed (**Supplementary Table 11**). Functional analysis upon differentially-expressed genes suggest the existence of many biochemical pathways that could be activated at different levels in the different species (**Figure 7**). For example, phosphate transport is more active in *C. × sinensis* than in *M. paniculata*, and genes related to cellular response to phosphate starvation are more expressed in *B. koenigii* than in *C. × sinensis*, whereas genes related to the serine metabolic pathway or the ‘tricarboxylic acid cycle’ are more expressed in *C. × sinensis* than in *B. koenigii*. The complete list of categories is shown in **Supplementary Table 12**. These results suggest that the biological composition of the phloem sap from the flushes of the three species is very different and could also be contributing to their distinct response to *C*Las inoculation.

## Discussion

The current model of plant resistance settles mostly on studies of mesophyll-infecting pathogens, and perhaps its most relevant feature is the activation of inducible responses by the plant upon pathogen recognition (Zhou and Zhang, 2020). Therefore, it is not surprising that many transcriptomic studies designed to evaluate the responses to *C*Las in susceptible or tolerant *Citrus* species centered the discussions on the deployment or not of complete defensive responses (Arce-Leal et al., 2020; Weber et al., 2022; Li et al., 2024). To our understanding, two essential aspects have been overlooked in many of these studies. On one hand, if *C*Las recognition by tolerant plants leads to PTI or ETI, a transcriptomic response should be activated very early after bacteria recognition. The foundation of the deployment of a robust PTI lies in the activation of stress-responsive transcription factors and the gene networks under their control, which begins soon after pathogen recognition (Li et al., 2020a). PTI is rapidly evaded by successful pathogens through the action of secreted effectors acting into the plant cells. When these effectors are recognized by resistant plans, ETI is activated, characterized by a marked transcriptional response within hours after pathogen recognition (Mine et al., 2018). Omics studies on HLB symptomatic samples, in which *C*Las infection took place months earlier, are not targeting the first molecular events after *C*Las recognition. In addition, *C*Las-infected material inoculated by grafting requires the earliest time point to be sampled weeks after inoculation to allow vascular recovery and *C*Las translocation to become detectable by qPCR (Raiol-Junior et al., 2021). Consequently, it is also an inadequate experimental system to evaluate early events of *C*Las infection. Simulation of natural entry by ACP-mediated inoculation using young shoots (Alves et al., 2021a; Lopes and Cifuentes-Arenas, 2021), as the one used by Wei et al. (2021) or in this work, seems to be more realistic to elucidate the molecular events taking place in this plant-pathogen interaction.

On the other hand, a complete immune response involves a massive transcriptional reprogramming of the cell, in the range of thousands of differentially-expressed genes (Lewis et al., 2015; Li et al., 2016; Mine et al., 2018). For example, in Arabidopsis, up to 25% of the genes presents altered gene expression after inoculation with the virulent *Pseudomonas syringae* (Tao et al, 2003), and in *Citrus*, more than 6000 genes were differentially expressed in the citrus canker resistant Kumquat 72 hours after recognition of *Xanthomonas citri* subs. *citri*, its causal agent (Ferrara et al., 2020). Therefore, up-regulation of some defense-related genes does not necessarily mean that a complete defense response has been deployed.

The relatively low number of DEGs found in the early stages after pathogen inoculation into the susceptible *C. × sinensis* observed in this study aligns with other reports analyzing the response to *C*Las in asymptomatic plants. For example, Fan et al. (2012) reported no significant changes in gene expression at 5 weeks after grafting inoculation to either *C. × limon* or *C. × sinensis.* Similarly, Ramsey et al. (2020) identified only 313 DEGs at 2 weeks after grafting inoculation to *C. × limon*, and Lombardi et al. (2024) found only 223 DEGs 4 weeks after inoculation to *C. × sinensis*, this last report using also ACP-vectored inoculation. Interestingly, a limited transcriptional reprogramming is also observed in response to other phloem-infecting pathogens of citrus, such as *Spiroplasma citri* (McNeil et al., 2023), suggesting that this might be a common feature of vascular pathogens that share similar infection pathways. By contrast, Wei et al. (2021) detected a high number of DEGs as early as 1 day after *C*Las inoculation in ‘Valencia’ sweet orange.

Our results suggest the idea that exposure of susceptible plants to *C*Las is not dramatically altering gene expression and indicate that a canonical and complete transcriptional immune response is not being activated in this susceptible host, although the possibility of waves of transcriptional changes occurring between the time intervals used in this experimental design cannot be discarded. Two non-exclusive hypotheses could explain these observations: a) the secretion of bacterial effectors suppress PTI, as it is proposed for several proteins putatively secreted by *C*Las to suppress PTI (Clark et al., 2018; Shi et al., 2019; Clark et al., 2020; Basu et al., 2022; Shi et al., 2023a, Shi et al., 2023b). So far, the mechanism for secretion is unknown, as *C*Las does not have the main machinery for effector secretion present in most plant pathogens (Cui et al., 2015; Jain et al., 2015). These effectors could be part of an orchestrated strategy in which *C*Las could also secrete other proteins that may interfere with specific plant metabolites produced by nucleated cells, altering plant cell homeostasis for its own benefit, and finally inducing symptoms, as observed for other phloem-limited pathogens (MacLean et al., 2011). b) *C*Las remains unnoticed for mesophyll cells, due to the apoplastic entry and exclusive vascular localization mediated by ACP, and the nuclei-free sieve element are unable to mount an effective immune response (Jiang et al., 2019; Huang et al., 2020; Lewis et al., 2022).

The use of resistant species such as *M. paniculata* and *B. koenigii*, in which *C*Las establishment is clearly limited (Alves et al., 2021a) opens the possibility to explore the molecular basis for resistance. Monoembryonic plants may exhibit genetic differences attributable to seed propagation, which might influence gene expression profiles. However, both Aurantioideae species were introduced into Brazil many decades ago, are seed-propagated and widely used as ornamentals, being highly homozygous (Nguyen et al., 2019; our unpublished results). In any case, as observed in the PCA, samples for each time point were closely grouped, indicating consistency among samples. Surprisingly, the limited number of DEGs observed in these two interactions in the first weeks after ACP-mediated bacterial inoculation, as well as the lack of immune defense categories enriched after functional analysis, suggest that canonical PTI or ETI responses are not the main mechanisms responsible for resistance in these two species. However, functional analysis revealed that the bacterium was not completely evading transcriptional responses in the plants. Both *M. paniculata* and *B. koenigii* tissues react to the presence of the bacterium by reprogramming the transcriptome of basic cellular functions. In both species, *C*Las presence provoked a subtle but consistent decrease in the expression of photosynthesis-related genes, photosynthesis being an enriched category upon less-expressed genes as soon as 48 hours after exposure to the bacterium. Enrichment of photosynthesis-related categories after transcriptomic analysis has been reported upon *C*Las infection in susceptible plants (Albrecht and Bowman, 2008; Zhong et al., 2015; Hu et al., 2017; Liu et al., 2019), but these experiments were performed in symptomatic plants, in which starch accumulation had already impaired photosynthetic processes and induced chloroplast degradation (Etxeberria et al., 2009). As mentioned before, this transcriptomic signature can be considered an effect of visual symptoms and therefore unrelated to the events investigated here. A decrease in photosynthesis in *M. paniculata* and *B. koenigii* at these very early stages after *C*Las inoculation, however, could be perceived as a mechanism triggered by the plants to hamper pathogen colonization. Curiously, this was also observed by Wei et al. (2021) in sweet orange at 5 days post inoculation with *C*las-infected psyllids. Photosynthesis reduction has been reported in the incompatible interaction between tobacco and *Phytophthora nicotianae* (Scharte et al., 2005), as well as in resistant barley plants after inoculation with powdery mildew (Swarbrick et al., 2006). In *M. paniculata* or *B. koenigii*, a decrease in photosynthesis in the early stages of *C*Las-infection could be a mechanism to regulate source/sink relationships to limit pathogen spread (McIntyre et al., 2021). *C*Las moves from source to sink tissues through the phloem vessels. After inoculation, *C*Las remains in the young flushes until they are mature enough to become source tissues (Raiol-Junior et al., 2017; Alves et al., 2021a). In citrus, young flushes are nutritionally richer than mature flushes, with higher concentrations of total amino acids in the phloem sap, positively associated with *C*Las and ACP fitness (Setamou et al., 2016, Setamou et al., 2017). In this study, *C. × sinensis* flush maturation took longer than in *M. paniculata* and *B. koenigii*, and *C. × sinensis* flushes exposed to *C*Las-positive psyllids reached maturity slower than those exposed to *C*Las-negative psyllids. The combination of these two factors would increase the time window in which *C*Las have the available nutrients crucial for its establishment. However, in young, infected flushes of *M. paniculata* or *B. koenigii*, photosynthesis decay could translate into a retardation in the sink to source transition, compromising the establishment of *C*Las in hosts presenting nutrient inadequacy (Killiny, 2016). In this regard, the observation at t10 that two out of five *B. koenigii* samples exposed to *C*Las-positive psyllids cluster to samples exposed to t10 *C*Las-negative psyllids might be events in which the bacterium had failed to multiply in these plants.

Other biochemical processes altered after *C*Las exposure in *B. koenigii* could hamper bacterial survival and multiplication, such as the decrease in the biosynthesis of sulfur-containing amino acids. *C*Las is not able to synthesize cysteine *de novo* and must overcome its lack by acquiring it from the surrounding environment (Lai et al., 2016; Merfa et al., 2019). Interestingly, the expression of cysteine biosynthesis genes was also higher in non-inoculated *C. × sinensis* young flushes than in *Bergera*’*s* ones, suggesting that this metabolic constraint could be determinant for *C*Las multiplication in the resistant species. Although these cellular rearrangements are taking place upon *C*Las perception, the lack of nuclei in sieve elements suggests that these responses should be activated in adjacent nucleated cells. Similarly, in the case of *C. × sinensis* and other susceptible species, effector proteins, trafficking through the plasmodesmata (Cheval and Faulkner, 2018), could suppress immune signaling in nucleated cells and impact the production of systemic signals (Huang et al., 2024).

The lack of a *bona fide* defense response in *M. paniculata* and *B. koenigii* prompted us to investigate whether these species could also have physiological or metabolic constraints that could limit *C*Las establishment. Comparison of expression of orthologous genes provides an estimation of biological processes that are active in young flushes of any of the three species. Our analysis revealed that the three transcriptomes are very different, and consequently, their biochemical composition should also differ, as reported by Killiny (2016). The small genome content of *C*Las and the lack of core metabolic pathways indicate that this bacterium heavily depends on the host nutrition (Wang and Trivedi, 2013; Lai et al., 2016), which could impact on the suitability of different species as host. Our functional analysis suggest that the higher expression of genes related to phosphate transport in *C. × sinensis* or related to the response to phosphate starvation in *B. koenigii*, which is essential for Liberibacter optimal growth (Cruz-Munoz et al., 2019), differ between the three species. Moreover, *C. × sinensis* young flushes have a higher expression of genes of the tricarboxylic acid cycle (TCA) than *B. koenigii*. The ATP metabolism was also enriched upon genes more expressed in *C. × sinensis* than in *M. paniculata*. These findings likely reflect a higher activity of this metabolic pathway in the susceptible *C. × sinensis*. Citrate is the preferred carbon source for Liberibacter (Cruz-Munoz et al., 2018) and evidence suggests that *C*Las obtains energy by exploiting TCA cycle intermediates from the plant (Wang and Trivedi, 2013; Jain et al., 2017) and psyllid hosts (Killiny and Nehela, 2017). An ATP translocase has been identified in *C*Las, suggesting the potential to import ATP directly from their environment (Vahling et al., 2010). It could be hypothesized that a lower activity of the TCA cycle in *B. koenigii* and *M. paniculata* flushes could be determining their less suitability for *C*Las establishment. In the most extreme case of unsuitability for *C*Las survival, *B. koenigii* may be considered as a non-host of the bacterium.

Overall, our data support the hypothesis that most *C. × sinensis* cells do not recognize the presence of *C*Las after inoculation, likely due to the ACP strategy to introduce the bacterium directly into the phloem in less than two hours (Wu et al., 2016), which results in a very limited - transcriptional response. The fortuitous protection obtained by *C*Las in the vascular tissue would allow it to remain unnoticed for mesophyll cells, thus limiting the possibility to orchestrate a robust and effective defense response. This, together with the putative effector-mediated suppression of PTI would explain the long incubation period and the difficulty in managing this devastating disease. This model could also explain why foliar spray of inducers of pathogen resistance, such as salicylic acid, have proven to be ineffective in reducing bacterial titer in HLB-infected plants (Atwood and Brlansky, 2009; Li et al., 2021) as well as in other phloem-limited pathogens (Rodriguez-Saona et al., 2021). The same scheme would apply for *B. koenigii* and *M. paniculata*, although in these cases the presence of the bacterium in the phloem would induce biochemical rearrangements in the surrounding cells, suggesting the existence of signaling events initiated in the sieve elements upon *C*Las recognition.

## Data Availability

The datasets presented in this study can be found in online repositories. The names of the repository/repositories and accession number(s) can be found in the article/**Supplementary Material**. The datasets generated for this study can be found in the GEO database under the accession number GSE277583.

## Supplementary material

Supplementary Figure 1 Transcriptome changes in *Citrus* × *sinensis, Murraya paniculata* and *Bergera koenigii* flushes along the first 60 days after exposure to psyllids. **(A)** Number of down-regulated genes in *Citrus × sinensis* samples taken immediately (CLas_0, Ch_0), and after 10 (CLas_10, Ch_10), 20 (CLas_20, Ch_20), 30 (CLas_30, Ch_30) or 60 (CLas_60, Ch_60) days after exposure to *C*Las-negative (Ch, top) or *C*Las-positive (CLas, bottom) psyllids when compared against flushes before the exposure to psyllids (prior). **(B)** Number of down-regulated genes in *Murraya paniculata* samples taken immediately (MLas_0, Mh_0), and after 10 (MLas_10, Mh_10), 20 (MLas_20, Mh_20), 30 (MLas_30, Mh_30) or 60 (MLas_60, Mh_60) days after exposure to *C*Las-negative (Mh, top) or *C*Las-positive (MLas, bottom) psyllids when compared against flushes before the exposure to psyllids (prior). **(C)** Number of down-regulated genes in *Bergera koenigii* samples taken immediately (BLas_0, Bh_0), and after 10 (BLas_10, Bh_10), 20 (BLas_20, Bh_20), 30 (BLas_30, Bh_30) or 60 (BLas_60, Bh_60) days after exposure to CLas-negative (Bh, top) or CLas-positive (BLas, bottom) psyllids when compared against flushes before the exposure to psyllids (prior). *C*Las: *Candidatus* Liberibacter asiaticus. DEGs: Differentially expressed genes. In each graph, total number of DEGs (X axis) at each time point (Y axis) is shown in bottom-left panels. Intersection of sets of genes at multiple time points is shown in top right panels. Each column corresponds to a time point or set of time points (dots connected by lines below the X axis) containing the same DEGs. The time points shared are indicated in the graphic below the column, with the time points on the left.
