# Supplementary figures and images for: Transcriptomic analysis of early stages of ‘Candidatus Liberibacter asiaticus’ infection in susceptible and resistant species after inoculation by Diaphorina citri feeding on young shoots
Source: Front Plant Sci. 2025 Feb 20;16:1502953. doi: 10.3389/fpls.2025.1502953 (PMC11882604; doi:10.3389/fpls.2025.1502953)

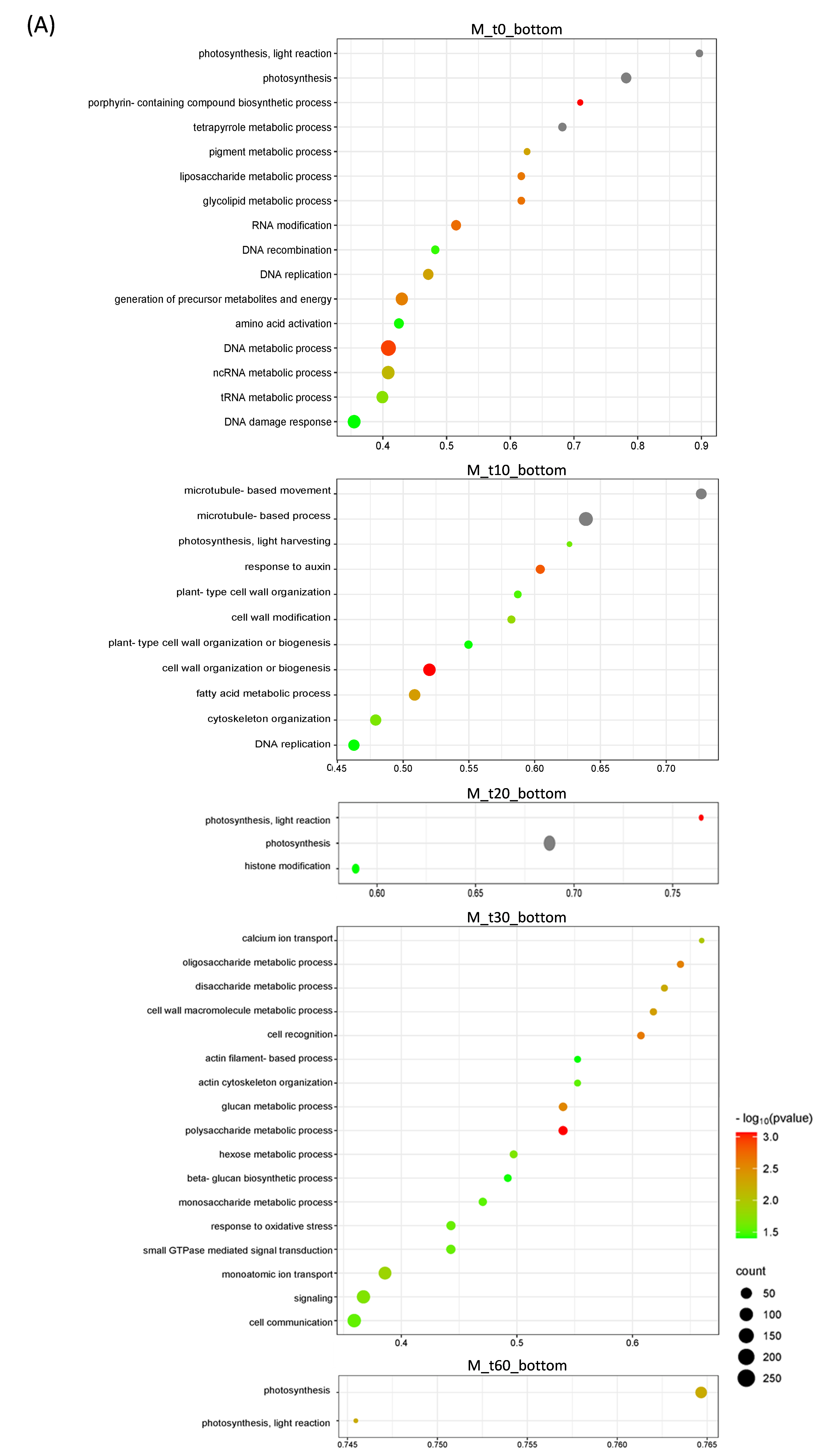

Supplement: Supplementary Figure 1 — Transcriptome changes in Citrus × sinensis, Murraya paniculata and Bergera koenigii flushes along the first 60 days after exposure to psyllids. (A) Number of down-regulated genes in Citrus × sinensis samples taken immediately (CLas_0, Ch_0), and after 10 (CLas_10, Ch_10), 20 (CLas_20, Ch_20), 30 (CLas_30, Ch_30) or 60 (CLas_60, Ch_60) days after exposure to CLas-negative (Ch, top) or CLas-positive (CLas, bottom) psyllids when compared against flushes before the exposure to psyllids (prior). (B) Number of down-regulated genes in Murraya paniculata samples taken immediately (MLas_0, Mh_0), and after 10 (MLas_10, Mh_10), 20 (MLas_20, Mh_20), 30 (MLas_30, Mh_30) or 60 (MLas_60, Mh_60) days after exposure to CLas-negative (Mh, top) or CLas-positive (MLas, bottom) psyllids when compared against flushes before the exposure to psyllids (prior). (C) Number of down-regulated genes in Bergera koenigii samples taken immediately (BLas_0, Bh_0), and after 10 (BLas_10, Bh_10), 20 (BLas_20, Bh_20), 30 (BLas_30, Bh_30) or 60 (BLas_60, Bh_60) days after exposure to CLas-negative (Bh, top) or CLas-positive (BLas, bottom) psyllids when compared against flushes before the exposure to psyllids (prior). CLas: Candidatus Liberibacter asiaticus. DEGs: Differentially expressed genes. In each graph, total number of DEGs (X axis) at each time point (Y axis) is shown in bottom-left panels. Intersection of sets of genes at multiple time points is shown in top right panels. Each column corresponds to a time point or set of time points (dots connected by lines below the X axis) containing the same DEGs. The time points shared are indicated in the graphic below the column, with the time points on the left. [file DataSheet1.zip › Figure 5A 180 mm.tif]

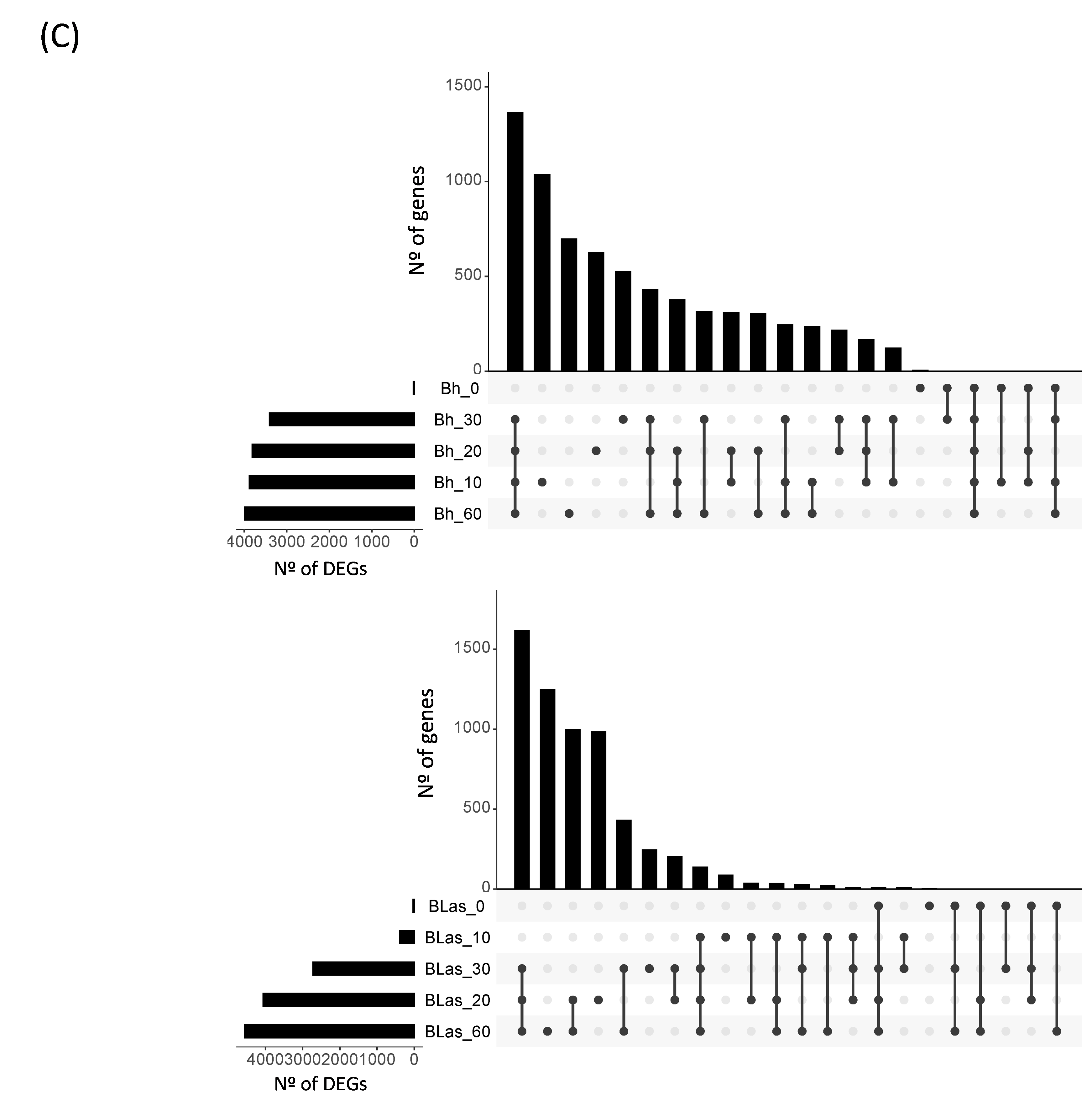

Supplement: Supplementary Figure 1 — Transcriptome changes in Citrus × sinensis, Murraya paniculata and Bergera koenigii flushes along the first 60 days after exposure to psyllids. (A) Number of down-regulated genes in Citrus × sinensis samples taken immediately (CLas_0, Ch_0), and after 10 (CLas_10, Ch_10), 20 (CLas_20, Ch_20), 30 (CLas_30, Ch_30) or 60 (CLas_60, Ch_60) days after exposure to CLas-negative (Ch, top) or CLas-positive (CLas, bottom) psyllids when compared against flushes before the exposure to psyllids (prior). (B) Number of down-regulated genes in Murraya paniculata samples taken immediately (MLas_0, Mh_0), and after 10 (MLas_10, Mh_10), 20 (MLas_20, Mh_20), 30 (MLas_30, Mh_30) or 60 (MLas_60, Mh_60) days after exposure to CLas-negative (Mh, top) or CLas-positive (MLas, bottom) psyllids when compared against flushes before the exposure to psyllids (prior). (C) Number of down-regulated genes in Bergera koenigii samples taken immediately (BLas_0, Bh_0), and after 10 (BLas_10, Bh_10), 20 (BLas_20, Bh_20), 30 (BLas_30, Bh_30) or 60 (BLas_60, Bh_60) days after exposure to CLas-negative (Bh, top) or CLas-positive (BLas, bottom) psyllids when compared against flushes before the exposure to psyllids (prior). CLas: Candidatus Liberibacter asiaticus. DEGs: Differentially expressed genes. In each graph, total number of DEGs (X axis) at each time point (Y axis) is shown in bottom-left panels. Intersection of sets of genes at multiple time points is shown in top right panels. Each column corresponds to a time point or set of time points (dots connected by lines below the X axis) containing the same DEGs. The time points shared are indicated in the graphic below the column, with the time points on the left. [file DataSheet1.zip › Figure 2C 180mm.tif]

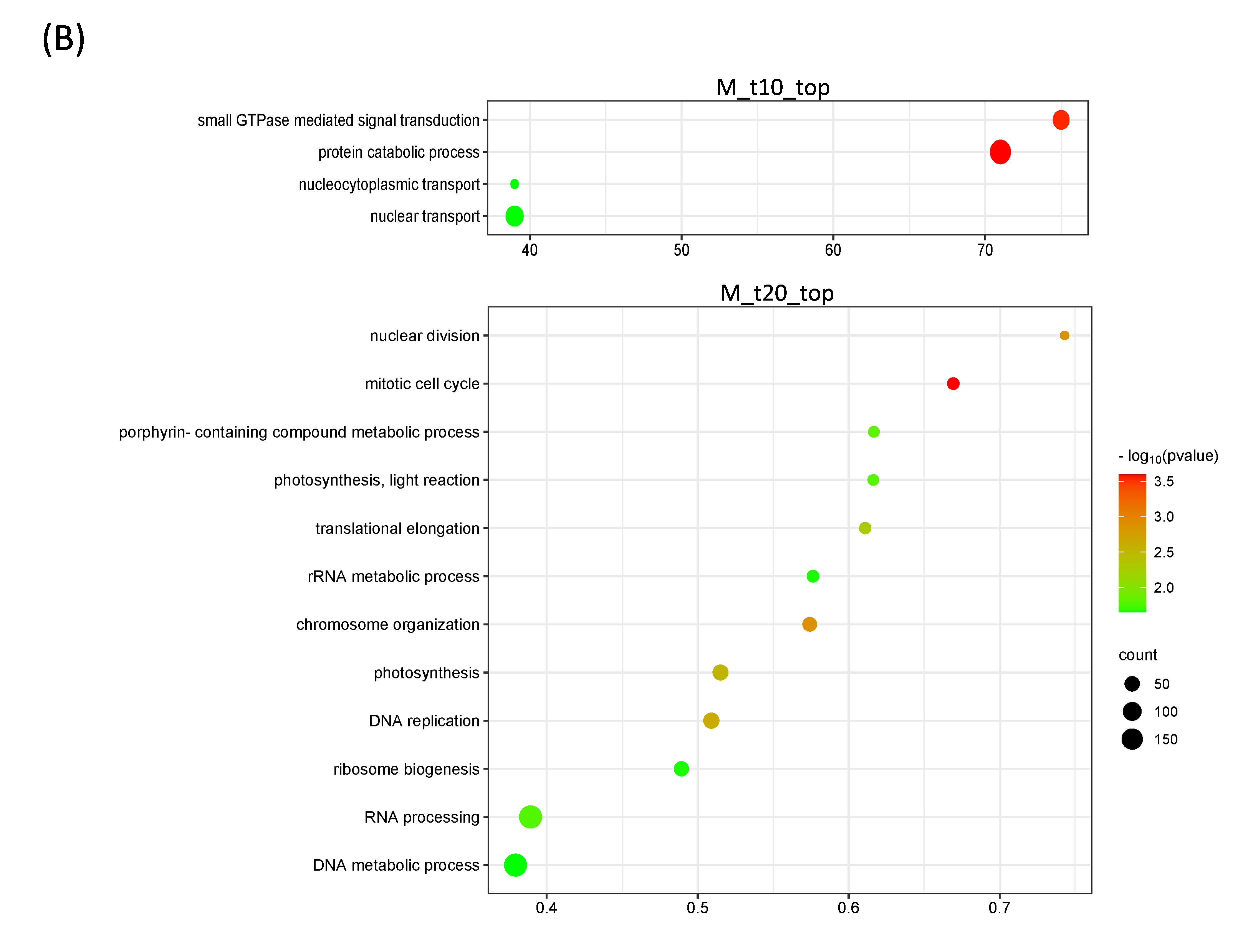

Supplement: Supplementary Figure 1 — Transcriptome changes in Citrus × sinensis, Murraya paniculata and Bergera koenigii flushes along the first 60 days after exposure to psyllids. (A) Number of down-regulated genes in Citrus × sinensis samples taken immediately (CLas_0, Ch_0), and after 10 (CLas_10, Ch_10), 20 (CLas_20, Ch_20), 30 (CLas_30, Ch_30) or 60 (CLas_60, Ch_60) days after exposure to CLas-negative (Ch, top) or CLas-positive (CLas, bottom) psyllids when compared against flushes before the exposure to psyllids (prior). (B) Number of down-regulated genes in Murraya paniculata samples taken immediately (MLas_0, Mh_0), and after 10 (MLas_10, Mh_10), 20 (MLas_20, Mh_20), 30 (MLas_30, Mh_30) or 60 (MLas_60, Mh_60) days after exposure to CLas-negative (Mh, top) or CLas-positive (MLas, bottom) psyllids when compared against flushes before the exposure to psyllids (prior). (C) Number of down-regulated genes in Bergera koenigii samples taken immediately (BLas_0, Bh_0), and after 10 (BLas_10, Bh_10), 20 (BLas_20, Bh_20), 30 (BLas_30, Bh_30) or 60 (BLas_60, Bh_60) days after exposure to CLas-negative (Bh, top) or CLas-positive (BLas, bottom) psyllids when compared against flushes before the exposure to psyllids (prior). CLas: Candidatus Liberibacter asiaticus. DEGs: Differentially expressed genes. In each graph, total number of DEGs (X axis) at each time point (Y axis) is shown in bottom-left panels. Intersection of sets of genes at multiple time points is shown in top right panels. Each column corresponds to a time point or set of time points (dots connected by lines below the X axis) containing the same DEGs. The time points shared are indicated in the graphic below the column, with the time points on the left. [file DataSheet1.zip › Figure 5B 180 mm.tif]

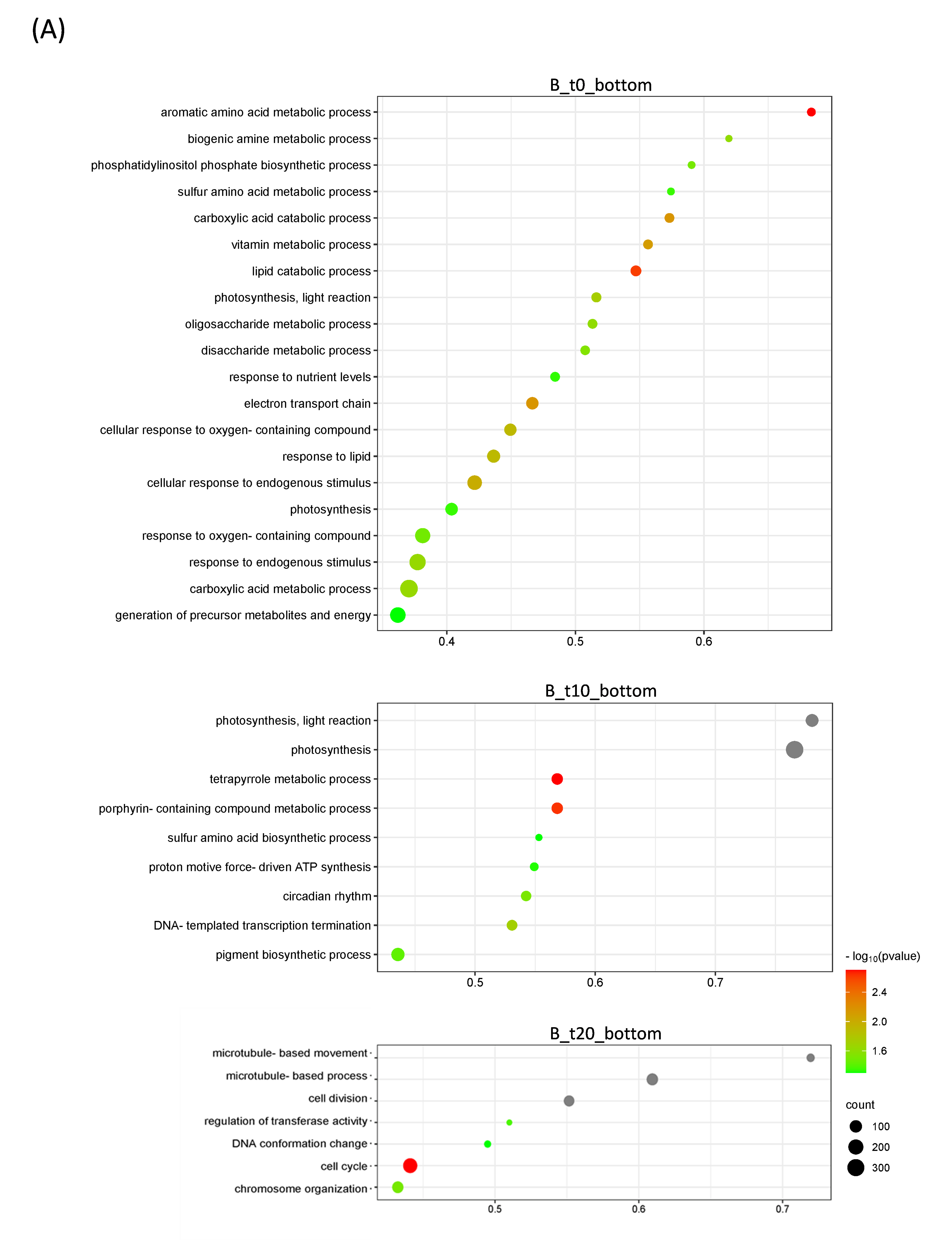

Supplement: Supplementary Figure 1 — Transcriptome changes in Citrus × sinensis, Murraya paniculata and Bergera koenigii flushes along the first 60 days after exposure to psyllids. (A) Number of down-regulated genes in Citrus × sinensis samples taken immediately (CLas_0, Ch_0), and after 10 (CLas_10, Ch_10), 20 (CLas_20, Ch_20), 30 (CLas_30, Ch_30) or 60 (CLas_60, Ch_60) days after exposure to CLas-negative (Ch, top) or CLas-positive (CLas, bottom) psyllids when compared against flushes before the exposure to psyllids (prior). (B) Number of down-regulated genes in Murraya paniculata samples taken immediately (MLas_0, Mh_0), and after 10 (MLas_10, Mh_10), 20 (MLas_20, Mh_20), 30 (MLas_30, Mh_30) or 60 (MLas_60, Mh_60) days after exposure to CLas-negative (Mh, top) or CLas-positive (MLas, bottom) psyllids when compared against flushes before the exposure to psyllids (prior). (C) Number of down-regulated genes in Bergera koenigii samples taken immediately (BLas_0, Bh_0), and after 10 (BLas_10, Bh_10), 20 (BLas_20, Bh_20), 30 (BLas_30, Bh_30) or 60 (BLas_60, Bh_60) days after exposure to CLas-negative (Bh, top) or CLas-positive (BLas, bottom) psyllids when compared against flushes before the exposure to psyllids (prior). CLas: Candidatus Liberibacter asiaticus. DEGs: Differentially expressed genes. In each graph, total number of DEGs (X axis) at each time point (Y axis) is shown in bottom-left panels. Intersection of sets of genes at multiple time points is shown in top right panels. Each column corresponds to a time point or set of time points (dots connected by lines below the X axis) containing the same DEGs. The time points shared are indicated in the graphic below the column, with the time points on the left. [file DataSheet1.zip › Figure 6A 180mm.tif]

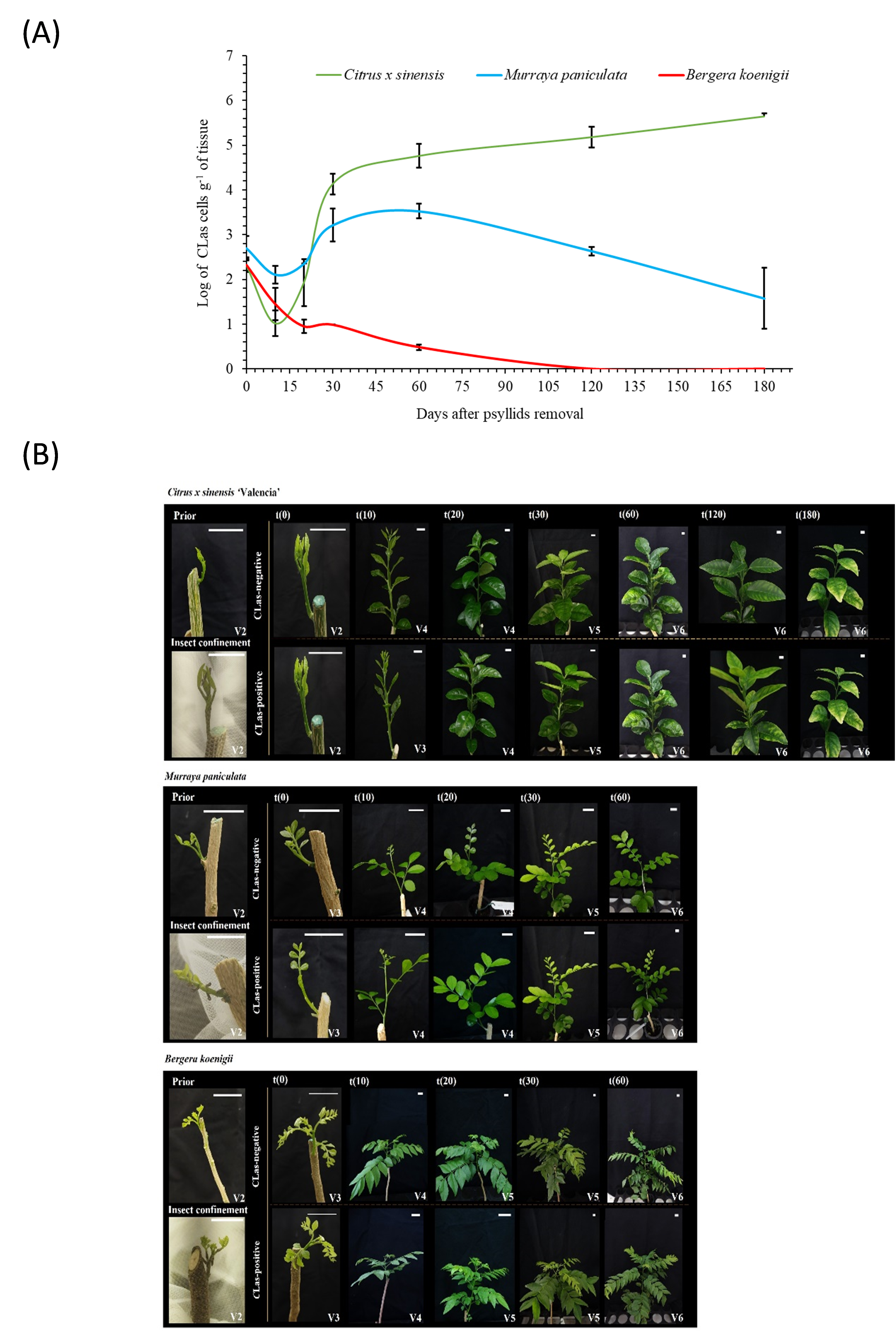

Supplement: Supplementary Figure 1 — Transcriptome changes in Citrus × sinensis, Murraya paniculata and Bergera koenigii flushes along the first 60 days after exposure to psyllids. (A) Number of down-regulated genes in Citrus × sinensis samples taken immediately (CLas_0, Ch_0), and after 10 (CLas_10, Ch_10), 20 (CLas_20, Ch_20), 30 (CLas_30, Ch_30) or 60 (CLas_60, Ch_60) days after exposure to CLas-negative (Ch, top) or CLas-positive (CLas, bottom) psyllids when compared against flushes before the exposure to psyllids (prior). (B) Number of down-regulated genes in Murraya paniculata samples taken immediately (MLas_0, Mh_0), and after 10 (MLas_10, Mh_10), 20 (MLas_20, Mh_20), 30 (MLas_30, Mh_30) or 60 (MLas_60, Mh_60) days after exposure to CLas-negative (Mh, top) or CLas-positive (MLas, bottom) psyllids when compared against flushes before the exposure to psyllids (prior). (C) Number of down-regulated genes in Bergera koenigii samples taken immediately (BLas_0, Bh_0), and after 10 (BLas_10, Bh_10), 20 (BLas_20, Bh_20), 30 (BLas_30, Bh_30) or 60 (BLas_60, Bh_60) days after exposure to CLas-negative (Bh, top) or CLas-positive (BLas, bottom) psyllids when compared against flushes before the exposure to psyllids (prior). CLas: Candidatus Liberibacter asiaticus. DEGs: Differentially expressed genes. In each graph, total number of DEGs (X axis) at each time point (Y axis) is shown in bottom-left panels. Intersection of sets of genes at multiple time points is shown in top right panels. Each column corresponds to a time point or set of time points (dots connected by lines below the X axis) containing the same DEGs. The time points shared are indicated in the graphic below the column, with the time points on the left. [file DataSheet1.zip › Fig 1 tiff 180 mm.tif]

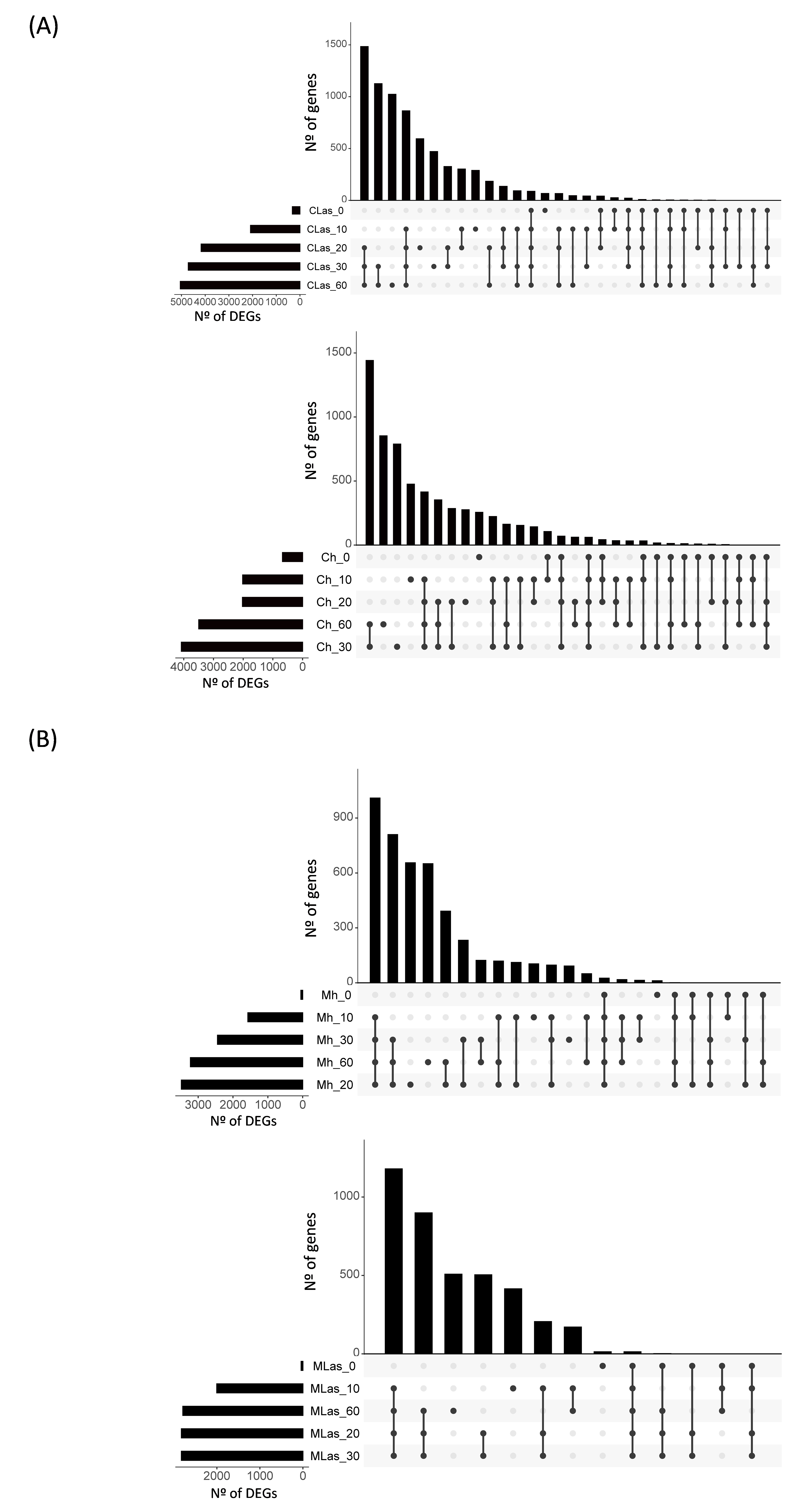

Supplement: Supplementary Figure 1 — Transcriptome changes in Citrus × sinensis, Murraya paniculata and Bergera koenigii flushes along the first 60 days after exposure to psyllids. (A) Number of down-regulated genes in Citrus × sinensis samples taken immediately (CLas_0, Ch_0), and after 10 (CLas_10, Ch_10), 20 (CLas_20, Ch_20), 30 (CLas_30, Ch_30) or 60 (CLas_60, Ch_60) days after exposure to CLas-negative (Ch, top) or CLas-positive (CLas, bottom) psyllids when compared against flushes before the exposure to psyllids (prior). (B) Number of down-regulated genes in Murraya paniculata samples taken immediately (MLas_0, Mh_0), and after 10 (MLas_10, Mh_10), 20 (MLas_20, Mh_20), 30 (MLas_30, Mh_30) or 60 (MLas_60, Mh_60) days after exposure to CLas-negative (Mh, top) or CLas-positive (MLas, bottom) psyllids when compared against flushes before the exposure to psyllids (prior). (C) Number of down-regulated genes in Bergera koenigii samples taken immediately (BLas_0, Bh_0), and after 10 (BLas_10, Bh_10), 20 (BLas_20, Bh_20), 30 (BLas_30, Bh_30) or 60 (BLas_60, Bh_60) days after exposure to CLas-negative (Bh, top) or CLas-positive (BLas, bottom) psyllids when compared against flushes before the exposure to psyllids (prior). CLas: Candidatus Liberibacter asiaticus. DEGs: Differentially expressed genes. In each graph, total number of DEGs (X axis) at each time point (Y axis) is shown in bottom-left panels. Intersection of sets of genes at multiple time points is shown in top right panels. Each column corresponds to a time point or set of time points (dots connected by lines below the X axis) containing the same DEGs. The time points shared are indicated in the graphic below the column, with the time points on the left. [file DataSheet1.zip › Suplemental Figure 1A-B 180.tif]

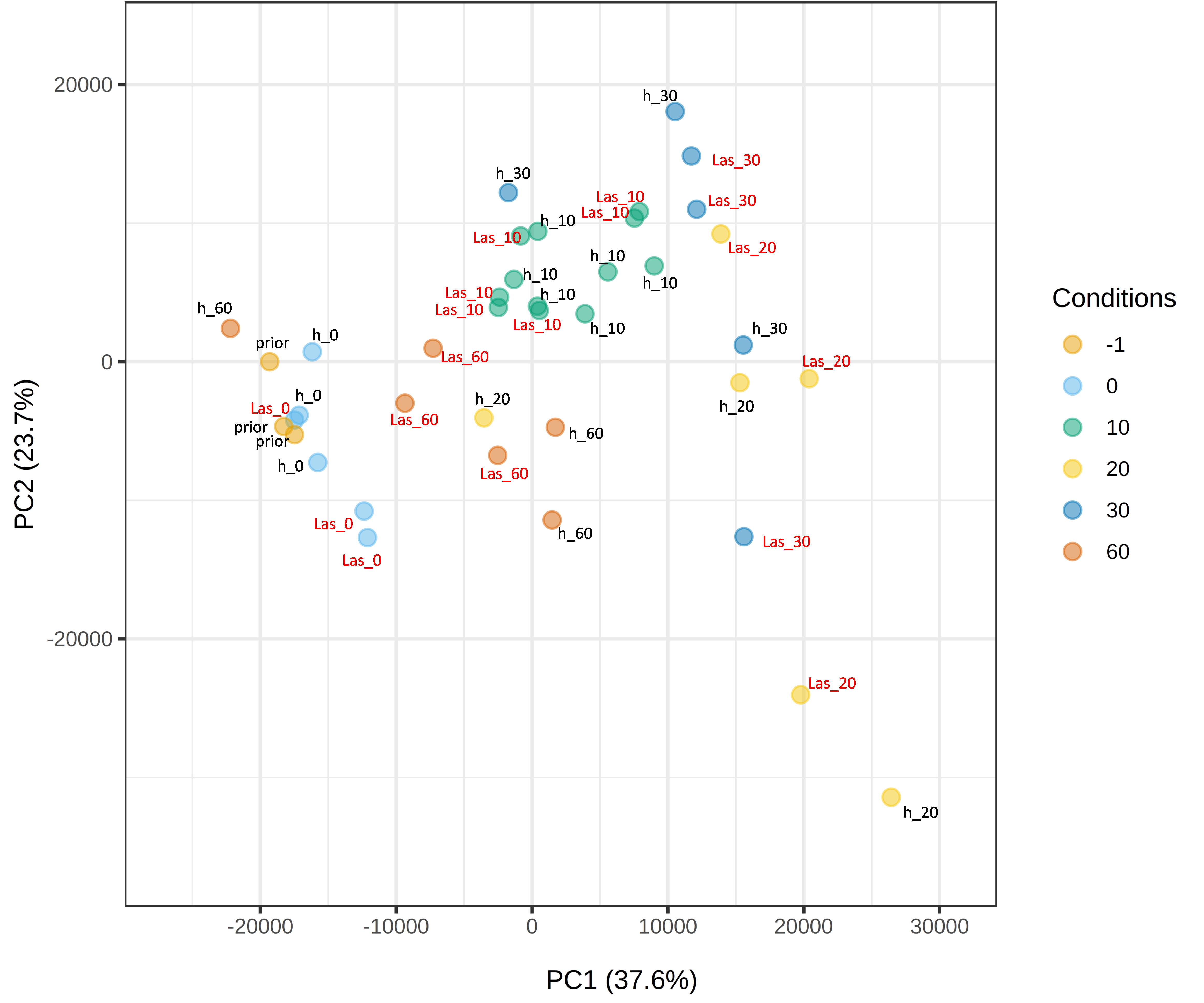

Supplement: Supplementary Figure 1 — Transcriptome changes in Citrus × sinensis, Murraya paniculata and Bergera koenigii flushes along the first 60 days after exposure to psyllids. (A) Number of down-regulated genes in Citrus × sinensis samples taken immediately (CLas_0, Ch_0), and after 10 (CLas_10, Ch_10), 20 (CLas_20, Ch_20), 30 (CLas_30, Ch_30) or 60 (CLas_60, Ch_60) days after exposure to CLas-negative (Ch, top) or CLas-positive (CLas, bottom) psyllids when compared against flushes before the exposure to psyllids (prior). (B) Number of down-regulated genes in Murraya paniculata samples taken immediately (MLas_0, Mh_0), and after 10 (MLas_10, Mh_10), 20 (MLas_20, Mh_20), 30 (MLas_30, Mh_30) or 60 (MLas_60, Mh_60) days after exposure to CLas-negative (Mh, top) or CLas-positive (MLas, bottom) psyllids when compared against flushes before the exposure to psyllids (prior). (C) Number of down-regulated genes in Bergera koenigii samples taken immediately (BLas_0, Bh_0), and after 10 (BLas_10, Bh_10), 20 (BLas_20, Bh_20), 30 (BLas_30, Bh_30) or 60 (BLas_60, Bh_60) days after exposure to CLas-negative (Bh, top) or CLas-positive (BLas, bottom) psyllids when compared against flushes before the exposure to psyllids (prior). CLas: Candidatus Liberibacter asiaticus. DEGs: Differentially expressed genes. In each graph, total number of DEGs (X axis) at each time point (Y axis) is shown in bottom-left panels. Intersection of sets of genes at multiple time points is shown in top right panels. Each column corresponds to a time point or set of time points (dots connected by lines below the X axis) containing the same DEGs. The time points shared are indicated in the graphic below the column, with the time points on the left. [file DataSheet1.zip › Figure 3 180 mm.tif]

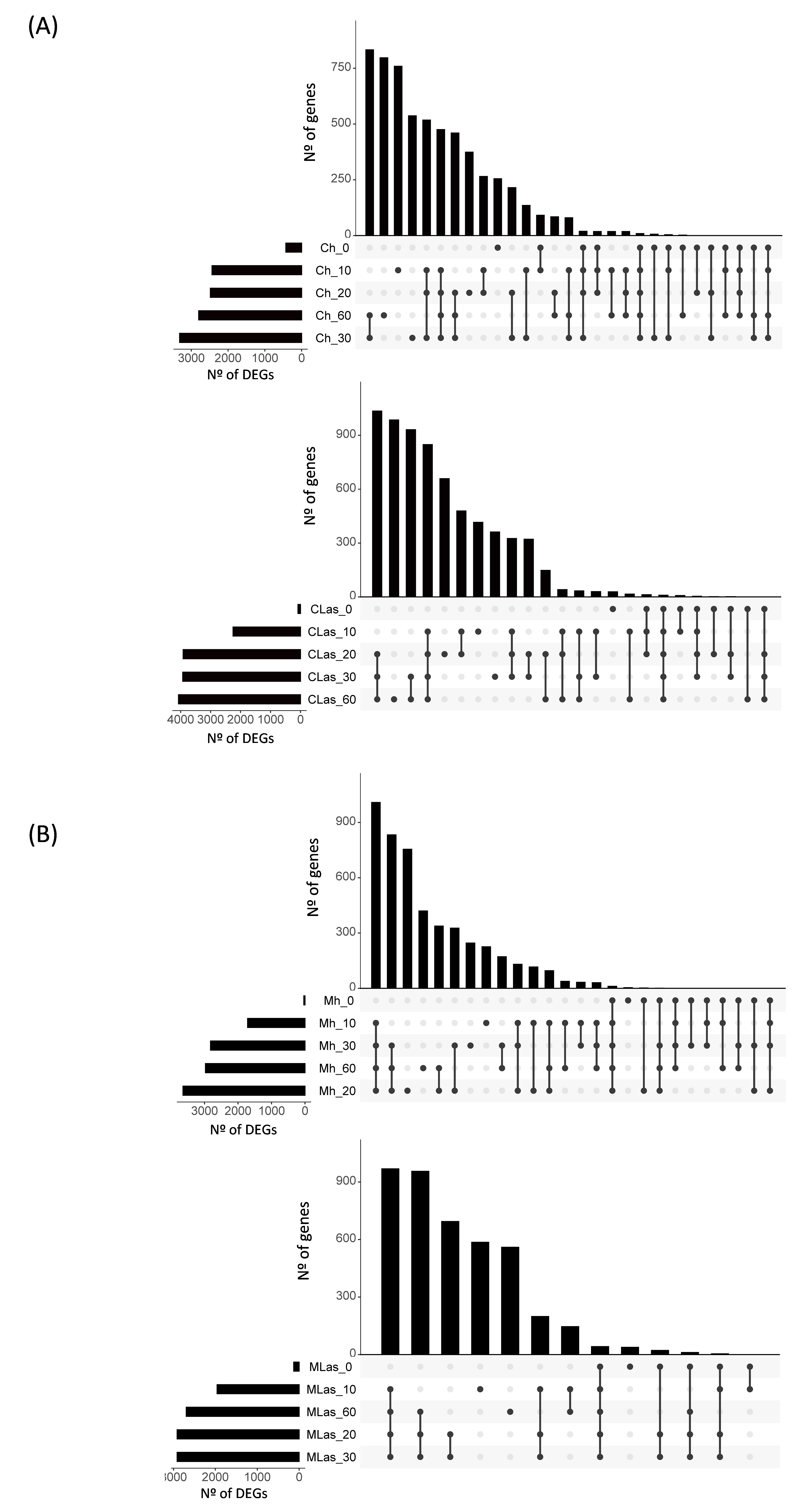

Supplement: Supplementary Figure 1 — Transcriptome changes in Citrus × sinensis, Murraya paniculata and Bergera koenigii flushes along the first 60 days after exposure to psyllids. (A) Number of down-regulated genes in Citrus × sinensis samples taken immediately (CLas_0, Ch_0), and after 10 (CLas_10, Ch_10), 20 (CLas_20, Ch_20), 30 (CLas_30, Ch_30) or 60 (CLas_60, Ch_60) days after exposure to CLas-negative (Ch, top) or CLas-positive (CLas, bottom) psyllids when compared against flushes before the exposure to psyllids (prior). (B) Number of down-regulated genes in Murraya paniculata samples taken immediately (MLas_0, Mh_0), and after 10 (MLas_10, Mh_10), 20 (MLas_20, Mh_20), 30 (MLas_30, Mh_30) or 60 (MLas_60, Mh_60) days after exposure to CLas-negative (Mh, top) or CLas-positive (MLas, bottom) psyllids when compared against flushes before the exposure to psyllids (prior). (C) Number of down-regulated genes in Bergera koenigii samples taken immediately (BLas_0, Bh_0), and after 10 (BLas_10, Bh_10), 20 (BLas_20, Bh_20), 30 (BLas_30, Bh_30) or 60 (BLas_60, Bh_60) days after exposure to CLas-negative (Bh, top) or CLas-positive (BLas, bottom) psyllids when compared against flushes before the exposure to psyllids (prior). CLas: Candidatus Liberibacter asiaticus. DEGs: Differentially expressed genes. In each graph, total number of DEGs (X axis) at each time point (Y axis) is shown in bottom-left panels. Intersection of sets of genes at multiple time points is shown in top right panels. Each column corresponds to a time point or set of time points (dots connected by lines below the X axis) containing the same DEGs. The time points shared are indicated in the graphic below the column, with the time points on the left. [file DataSheet1.zip › Figure 2A-B 180 mm.tif]

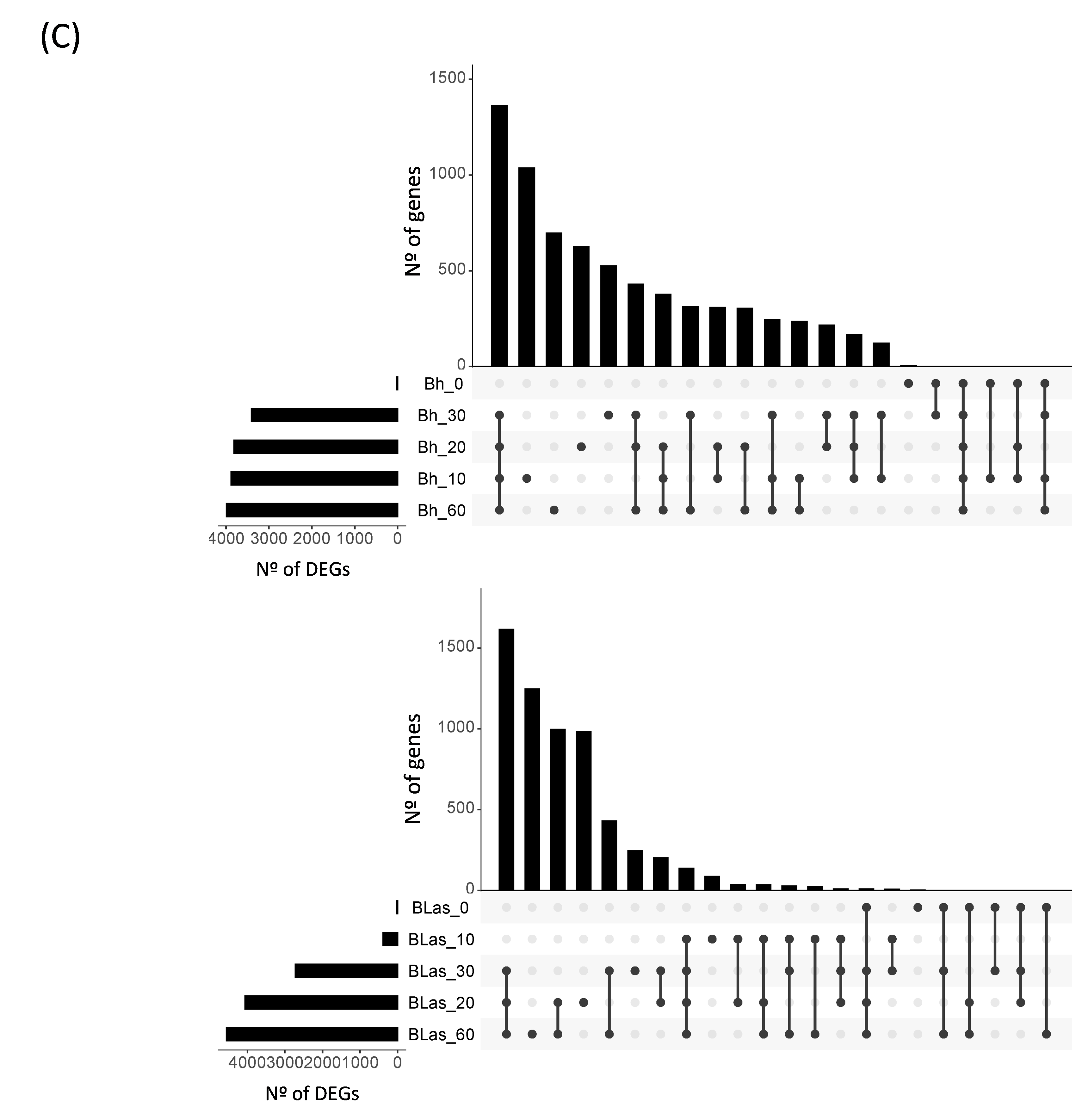

Supplement: Supplementary Figure 1 — Transcriptome changes in Citrus × sinensis, Murraya paniculata and Bergera koenigii flushes along the first 60 days after exposure to psyllids. (A) Number of down-regulated genes in Citrus × sinensis samples taken immediately (CLas_0, Ch_0), and after 10 (CLas_10, Ch_10), 20 (CLas_20, Ch_20), 30 (CLas_30, Ch_30) or 60 (CLas_60, Ch_60) days after exposure to CLas-negative (Ch, top) or CLas-positive (CLas, bottom) psyllids when compared against flushes before the exposure to psyllids (prior). (B) Number of down-regulated genes in Murraya paniculata samples taken immediately (MLas_0, Mh_0), and after 10 (MLas_10, Mh_10), 20 (MLas_20, Mh_20), 30 (MLas_30, Mh_30) or 60 (MLas_60, Mh_60) days after exposure to CLas-negative (Mh, top) or CLas-positive (MLas, bottom) psyllids when compared against flushes before the exposure to psyllids (prior). (C) Number of down-regulated genes in Bergera koenigii samples taken immediately (BLas_0, Bh_0), and after 10 (BLas_10, Bh_10), 20 (BLas_20, Bh_20), 30 (BLas_30, Bh_30) or 60 (BLas_60, Bh_60) days after exposure to CLas-negative (Bh, top) or CLas-positive (BLas, bottom) psyllids when compared against flushes before the exposure to psyllids (prior). CLas: Candidatus Liberibacter asiaticus. DEGs: Differentially expressed genes. In each graph, total number of DEGs (X axis) at each time point (Y axis) is shown in bottom-left panels. Intersection of sets of genes at multiple time points is shown in top right panels. Each column corresponds to a time point or set of time points (dots connected by lines below the X axis) containing the same DEGs. The time points shared are indicated in the graphic below the column, with the time points on the left. [file DataSheet1.zip › Suplemental Figure 1C 180.tif]

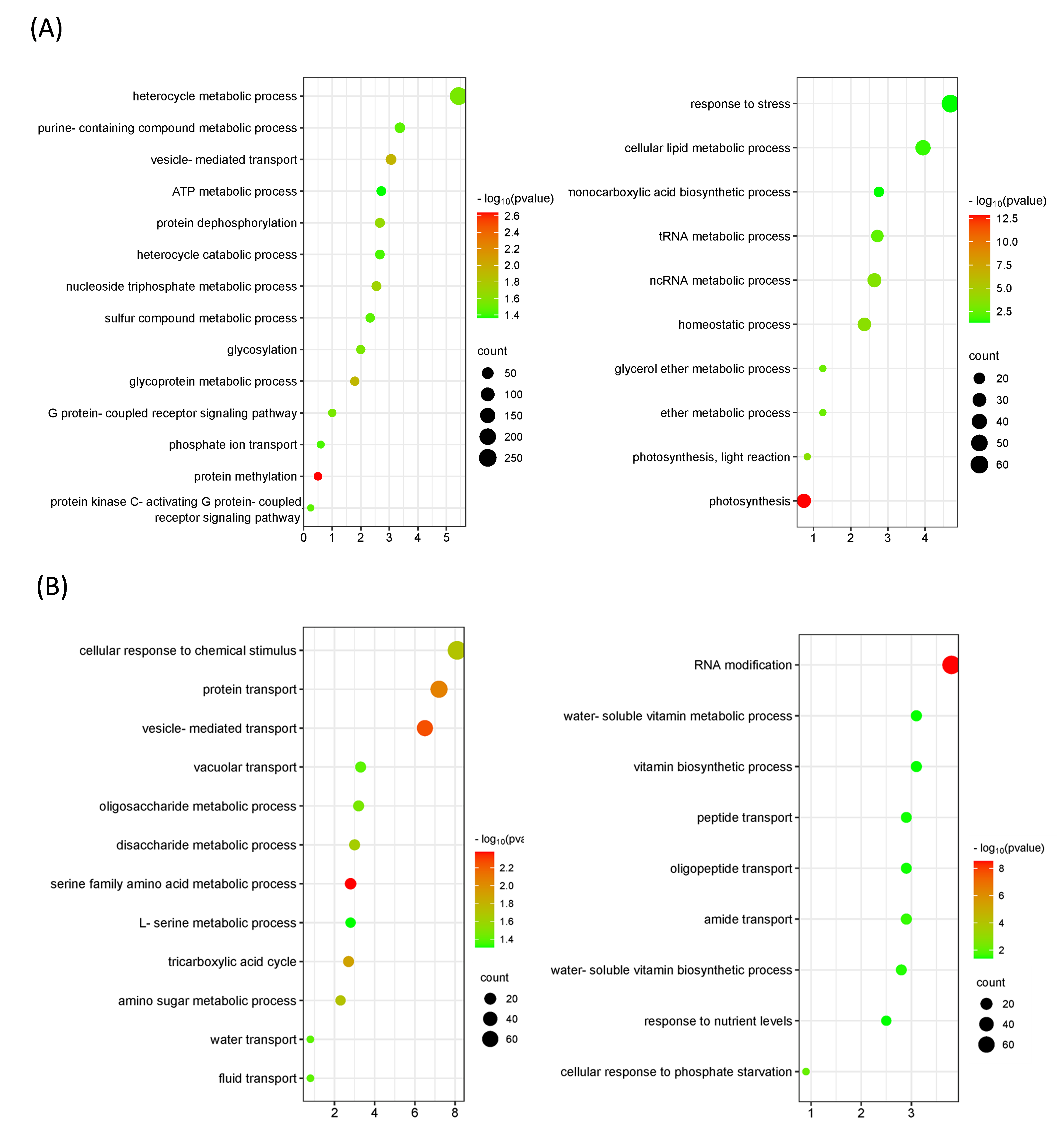

Supplement: Supplementary Figure 1 — Transcriptome changes in Citrus × sinensis, Murraya paniculata and Bergera koenigii flushes along the first 60 days after exposure to psyllids. (A) Number of down-regulated genes in Citrus × sinensis samples taken immediately (CLas_0, Ch_0), and after 10 (CLas_10, Ch_10), 20 (CLas_20, Ch_20), 30 (CLas_30, Ch_30) or 60 (CLas_60, Ch_60) days after exposure to CLas-negative (Ch, top) or CLas-positive (CLas, bottom) psyllids when compared against flushes before the exposure to psyllids (prior). (B) Number of down-regulated genes in Murraya paniculata samples taken immediately (MLas_0, Mh_0), and after 10 (MLas_10, Mh_10), 20 (MLas_20, Mh_20), 30 (MLas_30, Mh_30) or 60 (MLas_60, Mh_60) days after exposure to CLas-negative (Mh, top) or CLas-positive (MLas, bottom) psyllids when compared against flushes before the exposure to psyllids (prior). (C) Number of down-regulated genes in Bergera koenigii samples taken immediately (BLas_0, Bh_0), and after 10 (BLas_10, Bh_10), 20 (BLas_20, Bh_20), 30 (BLas_30, Bh_30) or 60 (BLas_60, Bh_60) days after exposure to CLas-negative (Bh, top) or CLas-positive (BLas, bottom) psyllids when compared against flushes before the exposure to psyllids (prior). CLas: Candidatus Liberibacter asiaticus. DEGs: Differentially expressed genes. In each graph, total number of DEGs (X axis) at each time point (Y axis) is shown in bottom-left panels. Intersection of sets of genes at multiple time points is shown in top right panels. Each column corresponds to a time point or set of time points (dots connected by lines below the X axis) containing the same DEGs. The time points shared are indicated in the graphic below the column, with the time points on the left. [file DataSheet1.zip › Figure 7 180 mm.tif]

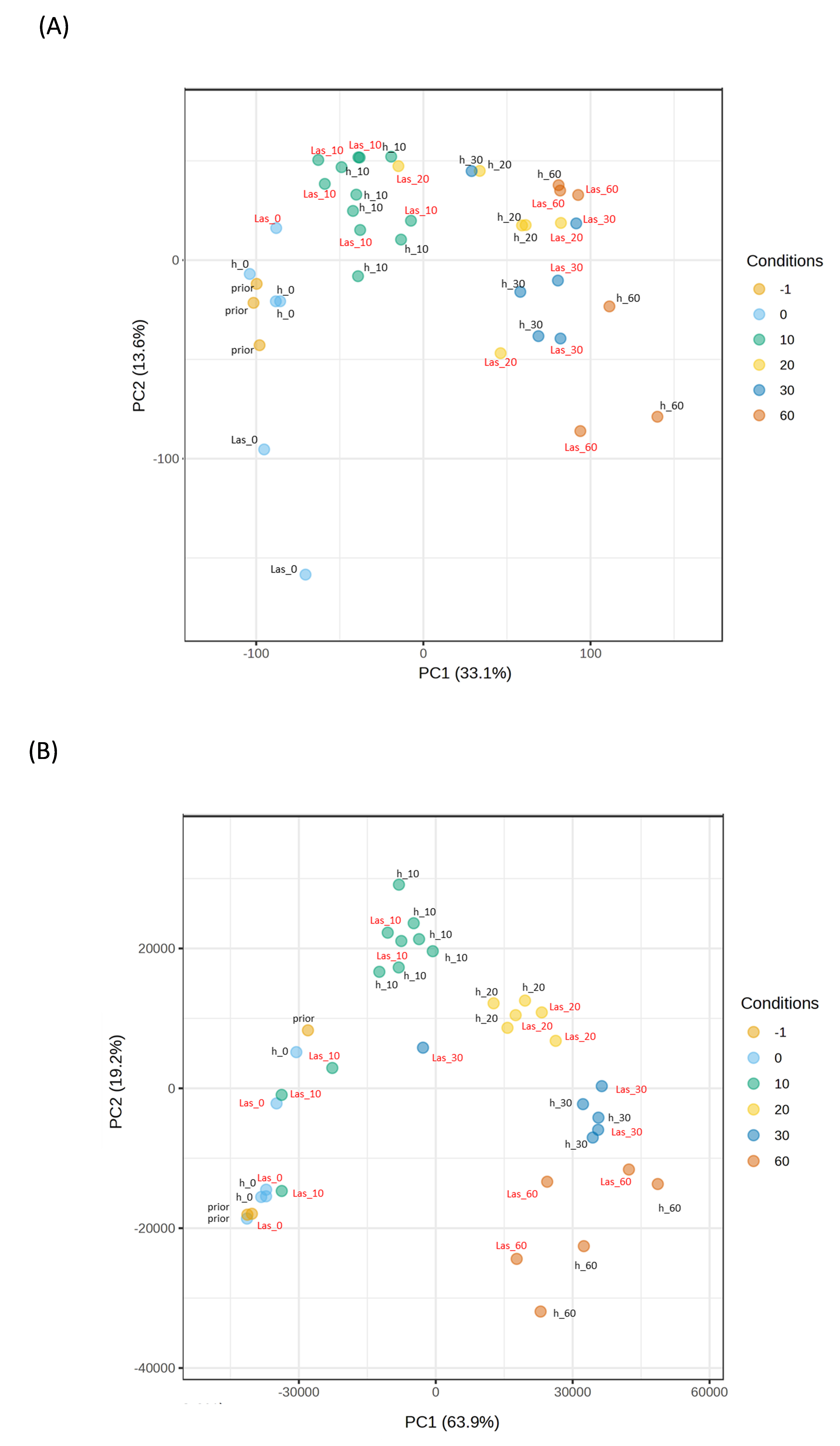

Supplement: Supplementary Figure 1 — Transcriptome changes in Citrus × sinensis, Murraya paniculata and Bergera koenigii flushes along the first 60 days after exposure to psyllids. (A) Number of down-regulated genes in Citrus × sinensis samples taken immediately (CLas_0, Ch_0), and after 10 (CLas_10, Ch_10), 20 (CLas_20, Ch_20), 30 (CLas_30, Ch_30) or 60 (CLas_60, Ch_60) days after exposure to CLas-negative (Ch, top) or CLas-positive (CLas, bottom) psyllids when compared against flushes before the exposure to psyllids (prior). (B) Number of down-regulated genes in Murraya paniculata samples taken immediately (MLas_0, Mh_0), and after 10 (MLas_10, Mh_10), 20 (MLas_20, Mh_20), 30 (MLas_30, Mh_30) or 60 (MLas_60, Mh_60) days after exposure to CLas-negative (Mh, top) or CLas-positive (MLas, bottom) psyllids when compared against flushes before the exposure to psyllids (prior). (C) Number of down-regulated genes in Bergera koenigii samples taken immediately (BLas_0, Bh_0), and after 10 (BLas_10, Bh_10), 20 (BLas_20, Bh_20), 30 (BLas_30, Bh_30) or 60 (BLas_60, Bh_60) days after exposure to CLas-negative (Bh, top) or CLas-positive (BLas, bottom) psyllids when compared against flushes before the exposure to psyllids (prior). CLas: Candidatus Liberibacter asiaticus. DEGs: Differentially expressed genes. In each graph, total number of DEGs (X axis) at each time point (Y axis) is shown in bottom-left panels. Intersection of sets of genes at multiple time points is shown in top right panels. Each column corresponds to a time point or set of time points (dots connected by lines below the X axis) containing the same DEGs. The time points shared are indicated in the graphic below the column, with the time points on the left. [file DataSheet1.zip › Figure 4 180 mm.tif]

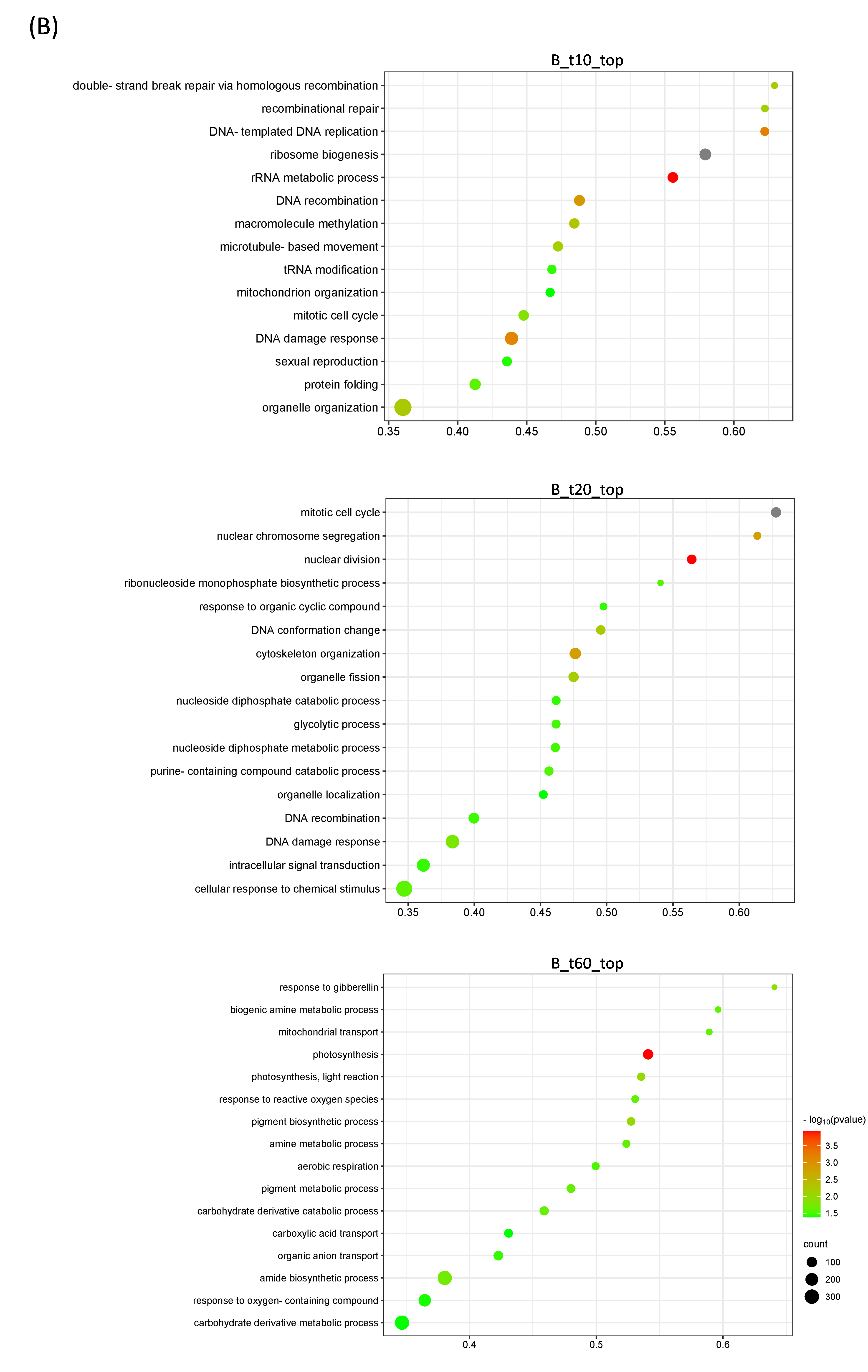

Supplement: Supplementary Figure 1 — Transcriptome changes in Citrus × sinensis, Murraya paniculata and Bergera koenigii flushes along the first 60 days after exposure to psyllids. (A) Number of down-regulated genes in Citrus × sinensis samples taken immediately (CLas_0, Ch_0), and after 10 (CLas_10, Ch_10), 20 (CLas_20, Ch_20), 30 (CLas_30, Ch_30) or 60 (CLas_60, Ch_60) days after exposure to CLas-negative (Ch, top) or CLas-positive (CLas, bottom) psyllids when compared against flushes before the exposure to psyllids (prior). (B) Number of down-regulated genes in Murraya paniculata samples taken immediately (MLas_0, Mh_0), and after 10 (MLas_10, Mh_10), 20 (MLas_20, Mh_20), 30 (MLas_30, Mh_30) or 60 (MLas_60, Mh_60) days after exposure to CLas-negative (Mh, top) or CLas-positive (MLas, bottom) psyllids when compared against flushes before the exposure to psyllids (prior). (C) Number of down-regulated genes in Bergera koenigii samples taken immediately (BLas_0, Bh_0), and after 10 (BLas_10, Bh_10), 20 (BLas_20, Bh_20), 30 (BLas_30, Bh_30) or 60 (BLas_60, Bh_60) days after exposure to CLas-negative (Bh, top) or CLas-positive (BLas, bottom) psyllids when compared against flushes before the exposure to psyllids (prior). CLas: Candidatus Liberibacter asiaticus. DEGs: Differentially expressed genes. In each graph, total number of DEGs (X axis) at each time point (Y axis) is shown in bottom-left panels. Intersection of sets of genes at multiple time points is shown in top right panels. Each column corresponds to a time point or set of time points (dots connected by lines below the X axis) containing the same DEGs. The time points shared are indicated in the graphic below the column, with the time points on the left. [file DataSheet1.zip › Figure 6-B 180 mm.tif]
